# A congenital CMV infection model for follow-up studies of neurodevelopmental disorders, neuroimaging abnormalities, and treatment

**DOI:** 10.1172/jci.insight.152551

**Published:** 2022-01-11

**Authors:** Yue-Peng Zhou, Meng-Jie Mei, Xian-Zhang Wang, Sheng-Nan Huang, Lin Chen, Ming Zhang, Xin-Yan Li, Hai-Bin Qin, Xiao Dong, Shuang Cheng, Le Wen, Bo Yang, Xue-Fang An, Ao-Di He, Bing Zhang, Wen-Bo Zeng, Xiao-Jun Li, Youming Lu, Hong-Chuang Li, Haidong Li, Wei-Guo Zou, Alec J. Redwood, Simon Rayner, Han Cheng, Michael A. McVoy, Qiyi Tang, William J. Britt, Xin Zhou, Xuan Jiang, Min-Hua Luo

**Affiliations:** 1 State Key Laboratory of Virology, CAS Center for Excellence in Brain Science and Intelligence Technology, Center for Biosafety Mega-Science, Wuhan Institute of Virology, Chinese Academy of Sciences, Wuhan, China.; 2 University of Chinese Academy of Sciences, Beijing, China.; 3 Hefei National Laboratory for Physical Sciences at the Microscale, School of Life Sciences, University of Science and Technology of China, Hefei, China.; 4 Key Laboratory of Magnetic Resonance in Biological Systems, State Key Laboratory of Magnetic Resonance and Atomic and Molecular Physics, National Center for Magnetic Resonance in Wuhan, Wuhan Institute of Physics and Mathematics, Innovation Academy of Precision Measurement Science and Technology, Chinese Academy of Sciences, Wuhan, China.; 5 The Institute for Brain Research, Collaborative Innovation Center for Brain Science, Huazhong University of Science and Technology, Wuhan, China.; 6 The Joint Center of Translational Precision Medicine, Guangzhou Institute of Pediatrics, Guangzhou Women and Children Medical Center, Guangzhou, China.; 7 State Key Laboratory of Cell Biology, CAS Center for Excellence in Molecular Cell Sciences, Shanghai Institute of Biochemistry and Cell Biology, Chinese Academy of Sciences, University of Chinese Academy of Sciences, Shanghai, China.; 8 The Institute for Respiratory Health, University of Western Australia, Crawley, Western Australia, Australia.; 9 Department of Medical Genetics, Oslo University Hospital and University of Oslo, Oslo, Norway.; 10 Hybrid Technology Hub — Centre of Excellence, Institute of Basic Medical Sciences, University of Oslo, Oslo, Norway.; 11 Shanghai Public Health Clinical Center, Fudan University, Shanghai, China.; 12 Department of Pediatrics, Virginia Commonwealth University School of Medicine, Richmond, Virginia, USA.; 13 Department of Microbiology, Howard University College of Medicine, Washington, DC, USA.; 14 Department of Pediatrics, School of Medicine, University of Alabama at Birmingham, Birmingham, Alabama, USA.

**Keywords:** Infectious disease, Virology, Mouse models, Neurodevelopment, Neuroimaging

## Abstract

Congenital cytomegalovirus (cCMV) infection is the leading infectious cause of neurodevelopmental disorders. However, the neuropathogenesis remains largely elusive due to a lack of informative animal models. In this study, we developed a congenital murine CMV (cMCMV) infection mouse model with high survival rate and long survival period that allowed long-term follow-up study of neurodevelopmental disorders. This model involves in utero intracranial injection and mimics many reported clinical manifestations of cCMV infection in infants, including growth restriction, hearing loss, and impaired cognitive and learning-memory abilities. We observed that abnormalities in MRI/CT neuroimaging were consistent with brain hemorrhage and loss of brain parenchyma, which was confirmed by pathological analysis. Neuropathological findings included ventriculomegaly and cortical atrophy associated with impaired proliferation and migration of neural progenitor cells in the developing brain at both embryonic and postnatal stages. Robust inflammatory responses during infection were shown by elevated inflammatory cytokine levels, leukocyte infiltration, and activation of microglia and astrocytes in the brain. Pathological analyses and CT neuroimaging revealed brain calcifications induced by cMCMV infection and cell death via pyroptosis. Furthermore, antiviral treatment with ganciclovir significantly improved neurological functions and mitigated brain damage as shown by CT neuroimaging. These results demonstrate that this model is suitable for investigation of mechanisms of infection-induced brain damage and long-term studies of neurodevelopmental disorders, including the development of interventions to limit CNS damage associated with cCMV infection.

## Graphical abstract

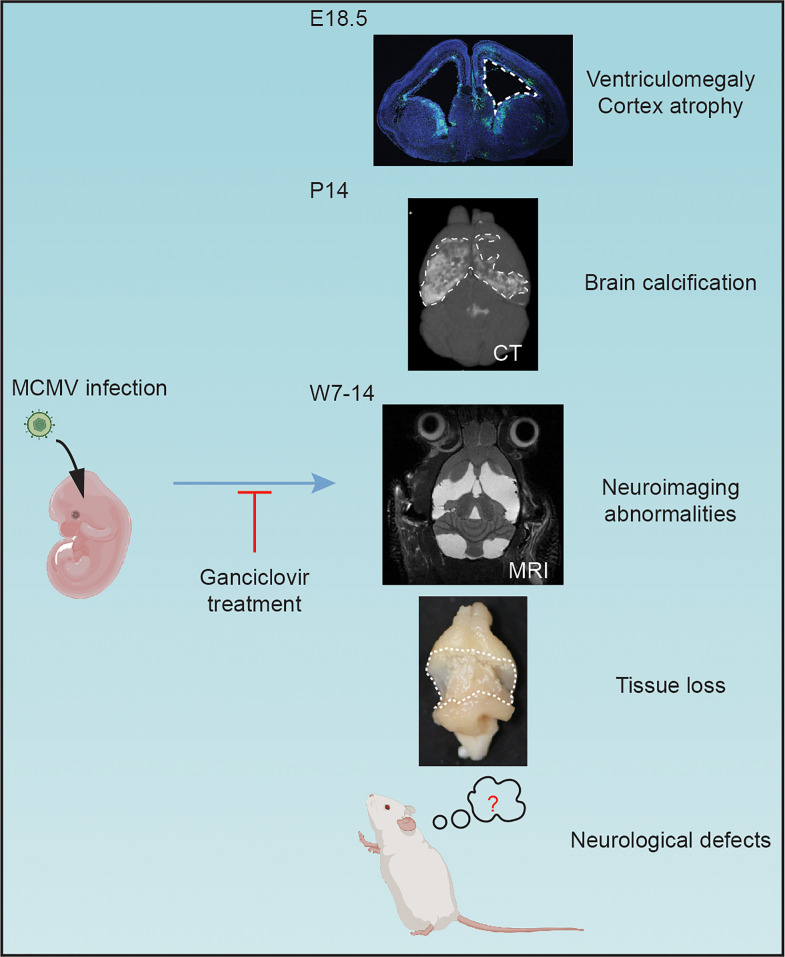

## Introduction

Congenital cytomegalovirus (cCMV) infection is the leading infectious cause of birth defects, primarily expressed as neurodevelopmental disorders (1). Estimates indicate that cCMV infection affects up to 0.64% of newborns globally (2). Between 5% and 10% of infected infants show symptoms at birth, manifested by hepatosplenomegaly, jaundice, petechial rash, brain calcification, or microcephaly. Additionally, 10% to 15% of asymptomatic infected infants subsequently develop late-onset sequelae during infancy or childhood, including sensorineural hearing loss (SNHL), intellectual disability, autism, and epilepsy (2). The association with CMV infection was identified in 1956 (3), but the neuropathogenic mechanisms that result in abnormal fetal brain development and long-term neurodevelopmental disorders remain poorly characterized. A major obstacle for addressing these important questions has been the lack of suitable animal models of cCMV infection to study long-term brain damage and associated neurodevelopmental disorders (4).

The fetal brain is a major target for end organ disease occurring during cCMV infection, and neural progenitor cells (NPCs) in fetal brains are highly susceptible to human CMV (HCMV) infection (4). Previous in vitro studies using primary human NPCs demonstrated that HCMV infection dysregulates NPC fate via diverse molecular mechanisms, including SOX2, Hes1, Notch1, and Jag1 in the Notch signaling pathway (5–7). Although these studies further our understanding of the mechanisms of HCMV neuropathogenesis, the in vitro NPC model cannot fully recapitulate structural brain damage and neurological defects induced by HCMV, and because mechanisms of disease identified in the NPC model require validation in vivo, an animal model suitable for studying neurodevelopmental damage induced by viral infection would be of considerable value.

As the strict species specificity of HCMV limits its study in experimental animals, guinea pig cytomegalovirus (GPCMV) (8, 9), rhesus macaque cytomegalovirus (10), and murine cytomegalovirus (MCMV) (11) models have been established. Given the high conservation of genome structure and the similar pathogenesis among the diverse CMV species, these animal models are well suited for congenital CMV infection in both small animals and nonhuman primates (12). Among these animal models, congenital MCMV (cMCMV) infection mouse models are one of the most commonly used to investigate the pathogenesis of cCMV infection as they offer several advantages: (i) MCMV infection shares similarities in pathology with HCMV infection; (ii) the genetic composition of the MCMV genome is similar to that of the HCMV genome; and (iii) perhaps most importantly, there are a large number of available resources, including reagents and transgenic mice (4). Existing mouse models established by either intracranial (i.c.) injection of MCMV into the ventricle of embryos during gestation or in the newborn period, or alternatively, intraperitoneal (i.p.) injection of newborns demonstrates a variety of neurodevelopmental disorders. These models have been successfully applied to identify a variety of neurodevelopmental disorders, including growth restriction, hydrocephaly, ventriculomegaly, and cortical atrophy at E18 (13, 14), and hearing loss, focal encephalitis, and abnormalities in cerebellar development in the postnatal period (15, 16).

To complement mouse models, a number of guinea pig models have been used to study vertical transmission, maternal protection, and vaccine development. For instance, a guinea pig model has been recently described that utilizes i.p. infection of newborn pups with GPCMV that results in neurodevelopmental disorder of spatial learning and memory at P15–P19 (8). All of these models provide insight into the mechanisms of HCMV-induced neurodevelopmental disorders in infants with cCMV infections by mimicking key symptoms, including hearing loss and neurological defects.

In the animal models described above, while the reported CMV-induced outcomes mimic the symptoms in natural cCMV infections, there are still certain outcomes, such as the brain calcification and abnormalities in neuroimaging, that are not captured. In addition, several models are limited by low survival rates of both infected embryos and newborn mice, limiting their value for long-term studies of neurodevelopment. Furthermore, several aspects of cCMV infection remain to be fully elucidated, including: (i) via serial studies, the long-term effects of cCMV infection on neurodevelopment from the early postnatal period until infected mice reach adult age; (ii) specific associations between brain damage and neurological defects, including behavioral changes; and (iii) more accurate correlations between pathological changes and neuroimaging findings of brain damage.

To investigate the mechanisms of cCMV infection associated with brain damage and neurological defects, we optimized the conditions for infection and developed a cMCMV infection mouse model for long-term follow-up studies in mice. Neurological defects and brain damage in this model were readily detected in infected mice up to 14 weeks postnatally. The brain damage revealed by abnormalities in MRI and CT neuroimaging were confirmed with pathological analysis. Neuroinflammation and cell death via pyroptosis and apoptosis were induced by MCMV infection, which was followed by brain calcification, a common finding described in brain tissues from fetuses and infants with cCMV infections (17). Treatment of infected mice with the antiviral drug ganciclovir (GCV) significantly improved neurological outcomes in MCMV-infected mice in this model. Taken together, this model displayed a series of characteristics that closely recapitulate the clinical manifestations of cCMV infection, making it suitable for exploration of long-term neurological defects induced by infection(s), including the development of treatments that could mollify the CNS damage associated with cCMV infection.

## Results

### A mouse model of cMCMV infection for follow-up studies: induction of long-term neurodevelopmental disorders, including hearing loss and impaired learning-memory ability.

**Figure 1:**
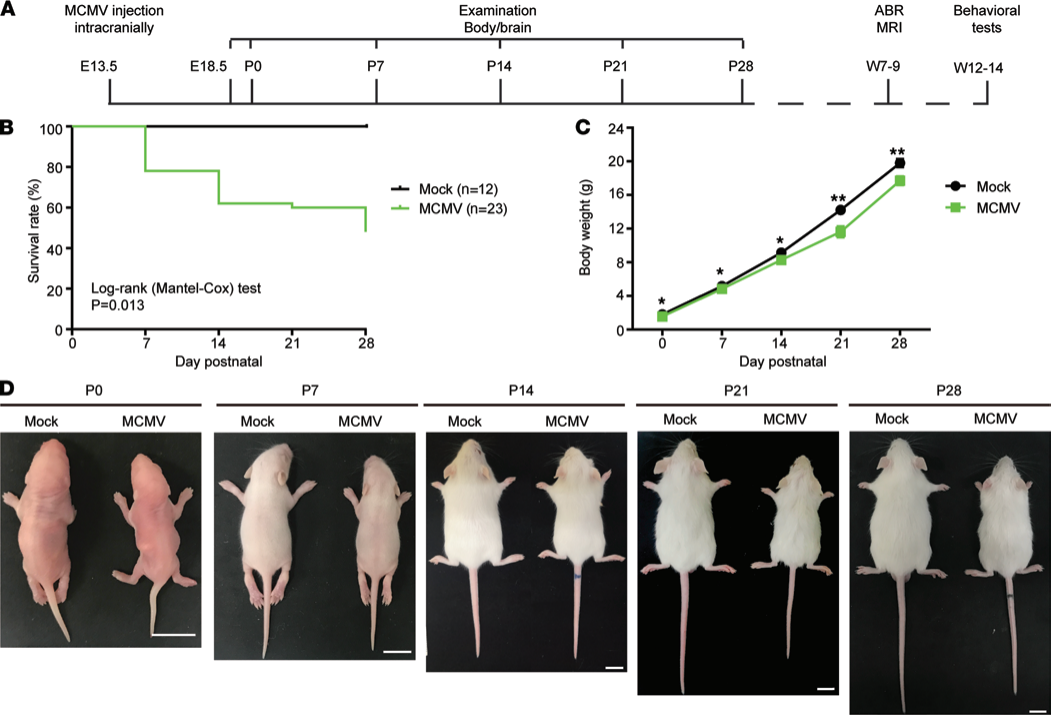
A cMCMV infection mouse model for follow-up studies of neurodevelopmental disorders. (**A**) Timeline. A dose of 20 PFU MCMV or equivalent conditioned media (Mock) was injected intracranially at E13.5. The bodies and brains of the mice were examined weekly from P0 to P28, or longer depending on experiments. Neuroimaging by MRI and auditory brainstem evoked response (ABR) tests were performed at 7–9 weeks postnatally (W7-9), and neurological tests were performed at W12–14. (**B**) Survival rates. Survival curves of mock- and MCMV-infected newborns from P0 to P28 are shown; the Mantel-Cox test was used for survival analysis. (**C**) Body weights. Body weights were measured weekly from P0 to P28. Data were analyzed by 2-tailed Student’s *t* test and results are presented as mean ± SEM. *, *P* < 0.05; **, *P* < 0.01. (**D**) General growth. Representative whole mouse images at the indicated time points are shown. Scale bar: 10 mm.

While i.p. inoculation of newborn mice is less technically challenging and results in CNS infection, this neurodevelopmental time point in mice corresponds to mid–second trimester human fetal development and therefore does not model first trimester HCMV infections that lead to most cases of severe structural damage in the CNS (18, 19). To more accurately represent early–first trimester cCMV infection, which is highly associated with severe neurological outcomes (20), a cMCMV infection mouse model was established by i.c. injection of recombinant MCMV (K181-eGFP strain) into the lateral ventricles of fetuses to initiate CMV infection in fetal brain at E13.5 (Figure 1A) (21). Inoculation at this time point allowed MCMV infection at a stage of CNS development equivalent to that of a first trimester human fetus (11). In a pilot study, i.c. inoculation of 1 × 10^5^ PFU of MCMV resulted in growth restriction with reduced body and brain weights (Supplemental Figure 1; supplemental material available online with this article; https://doi.org/10.1172/jci.insight.152551DS1), consistent with previous reports using the same viral dose (14). However, infection with such a high viral dose also resulted in a low survival rate of 16.9% (Table 1), which precluded long-term follow-up studies. After optimizing the viral dose (Table 1) and infection time (E12.5–E14.5, data not shown), we found that 20 PFU of MCMV at E13.5 could consistently induce neurological manifestations while ensuring a high survival rate at birth (~82%) and a long survival period (~65% survived at P14 and ~45% from P28 to W14). Mice that survived after P28 were used for neurodevelopmental studies, including neuroimaging, hearing, and other neurological tests W7 to W14 (Figure 1, A and B). Similar to findings from the high–viral dose model (Supplemental Figure 1), growth restriction with reduced body weights was observed in this optimized cMCMV infection mouse model (Figure 1, C and D).

**Figure 2:**
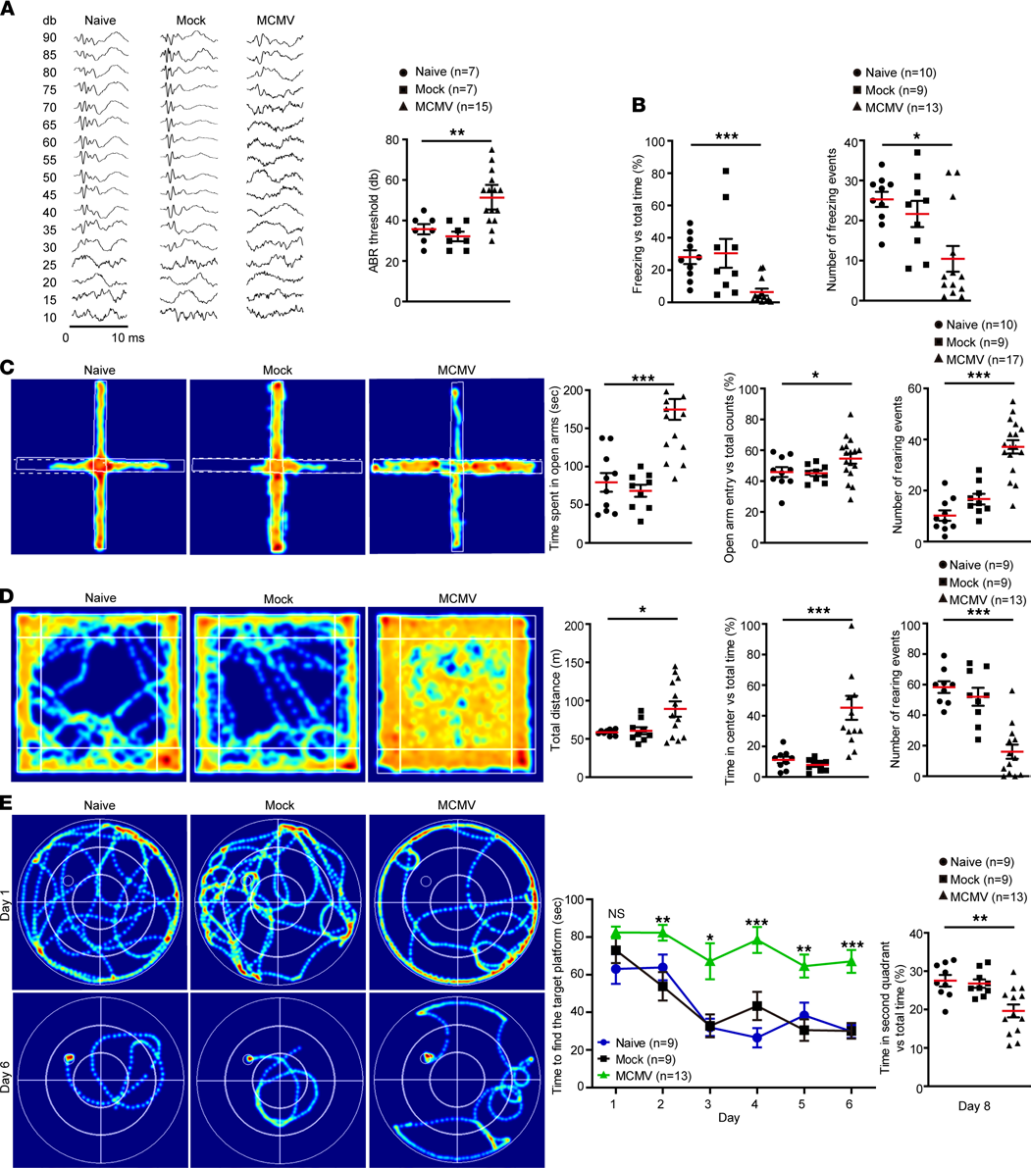
cMCMV infection induces auditory and behavioral abnormalities. (**A**) Auditory function. ABR was determined in naive and mock- or MCMV-infected mice at W7–8. Representative audiograms in 10 ms from naive and mock- or MCMV-infected pups tested over a range of frequencies (clicks). ABR thresholds are shown on the right. (**B**) Short-term learning-memory ability. Short-term learning-memory ability was assessed by fear conditioning test at W12–13, and proportions of freezing time and number of freezing events were recorded. (**C**) Anxiety-like behavior in EPMT. General anxiety to open spaces was evaluated at W12–13. Representative heatmaps of mouse movement tracks and durations are shown. Time spent in the open arms (indicated by white dashed line), percentage of entries to the open arms versus total counts, and number of rearing events were recorded. (**D**) Anxiety-like behavior in OFT. OFT was performed to assess anxiety-like behavior to open areas. Representative heatmaps of movement tracks and durations are shown. Total traveled distance, percentage of time traveled in the center, and number of rearing events were measured. (**E**) Spatial learning-memory. Spatial learning-memory abilities were determined by MWM test during W13–14. All mice were trained in the maze for 6 days and tested on the eighth day. Representative heatmaps of movement tracks and durations are shown. Total time of finding the target platform (indicated as a circle in the heatmap) from day 1 to day 6 and proportions of time in the second quadrant on day 8 were recorded. All data were analyzed by 1-way ANOVA test and results are presented as means ± SEM. *, *P* < 0.05; **, *P* < 0.01; ***, *P* < 0.001. Sample size *n* for each group is indicated.

Despite similar dietary habits and light/dark cycle activities (not shown), compared to naive and mock-infected control mice, MCMV-infected mice displayed neurodevelopmental disorders when tested between W7 and W14. ABR testing at W7–8 showed that the MCMV-infected mice presented an increased threshold value of approximately 50 dB as compared with the controls that had a threshold value of approximately 35 dB, indicating significant hearing loss (Figure 2A). In the cue and contextual fear conditioning test at W12–13, MCMV-infected mice displayed significantly shorter freezing duration and lower freezing frequency compared with controls, indicating an impaired short-term learning-memory ability (Figure 2B). Cognition of anxiety-like behavior was evaluated by an elevated plus maze test (EPMT) and open field test (OFT) at W12–13. EPMT showed that the MCMV-infected mice spent significantly more time in the open arm and entered the open arm more frequently than controls (Figure 2C). OFT showed that the MCMV-infected mice traveled a longer distance in the arena, especially in the center, and displayed longer exposure time in the open field than controls (Figure 2D). To assess spatial learning-memory ability, all the mice were trained for 6 days in W13 to locate a submerged platform in a Morris water maze (MWM), rested for 1 day, and tested at W14. The MCMV-infected mice showed a significant delay in learning and spent a lower proportion of time in the quadrant where the platform was located (Figure 2E). Taken together, these results demonstrate that cMCMV infection early in embryogenesis resulted in hearing loss, altered cognition, and impaired learning-memory ability. Each of these outcomes is consistent with the clinical manifestations of cCMV infection, suggesting this murine model is suitable for long-term follow-up studies of neurodevelopmental disorders associated with cCMV infections.

### cMCMV infection causes abnormalities in MRI neuroimaging and loss of brain parenchyma.

**Figure 3:**
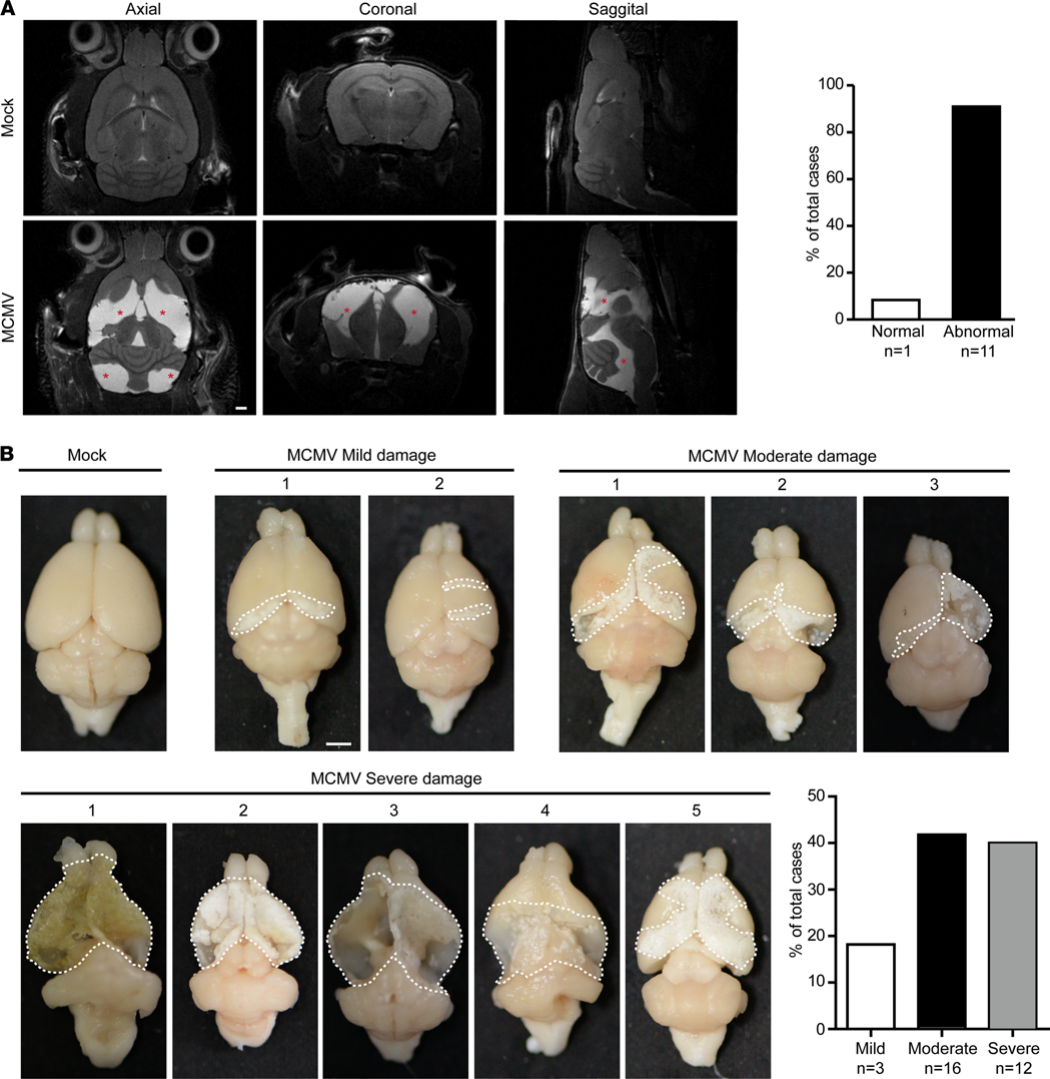
Altered neuroimaging findings and loss of brain parenchyma in infected mice. (**A**) Neuroimaging (anatomical imaging) by MRI. Brain damage was monitored by MRI at W8–9. Axial, sagittal, and coronal views of representative mock and MCMV-infected brains are shown, and abnormal hyperintense signals are indicated by red asterisks. In the MCMV-infected group the percentages of cases with or without abnormal hyperintense signals among the total cases were calculated. Sample size *n* is indicated. Scale bar: 1 mm. (**B**) Pathological examination of brain damage. Representative images of W14 mouse brains following neurological tests are shown. Damaged areas outlined by white dashed lines were measured. Brain damage was classified into mild (0%~30%), moderate (30%~70%), and severe (>70%) groups according to the percentage of damaged area to the total area of the brain. The proportions of cases in each group to total cases were calculated and presented. Sample size *n* is indicated. Scale bar: 2 mm.

To further understand the neurological manifestations in mice infected at an early stage in embryogenesis, we investigated cMCMV infection-induced brain changes by neuroimaging and pathological characterization. MRI, a noninvasive method, was performed at W8–9 after hearing assessment. Only the brains of the MCMV-infected mice (11 out of 12 randomly selected MCMV-infected mice) showed abnormal hyperintense signals in multiple areas in both the cerebrum and cerebellum (Figure 3A), suggesting cMCMV infection-induced brain damage can be detected by MRI at W8. Neuropathological studies were carried out in infected mice at W14. All specimens from the MCMV-infected animals displayed brain damage, with macroscopic lesions and substantial brain parenchyma loss observed mainly in the cortex (Figure 3B). The differences in the detection of damaged areas of the brain identified by these 2 methods may be due to different detection times (W8 vs. W14) or limitations in detection of pathological findings by MRI. Based on the severity of damage (percentage of the damaged area relative to the total cortex), brain damage was classified into mild (0%~30%), moderate (30%~70%), and severe (>70%). Among the MCMV-infected brains, more than 80% were categorized as having moderate and severe damage, indicating brain damage is a common feature of cMCMV infection in early embryogenesis.

### cMCMV infection causes ventriculomegaly and cortical atrophy in the developing brain.

**Figure 4:**
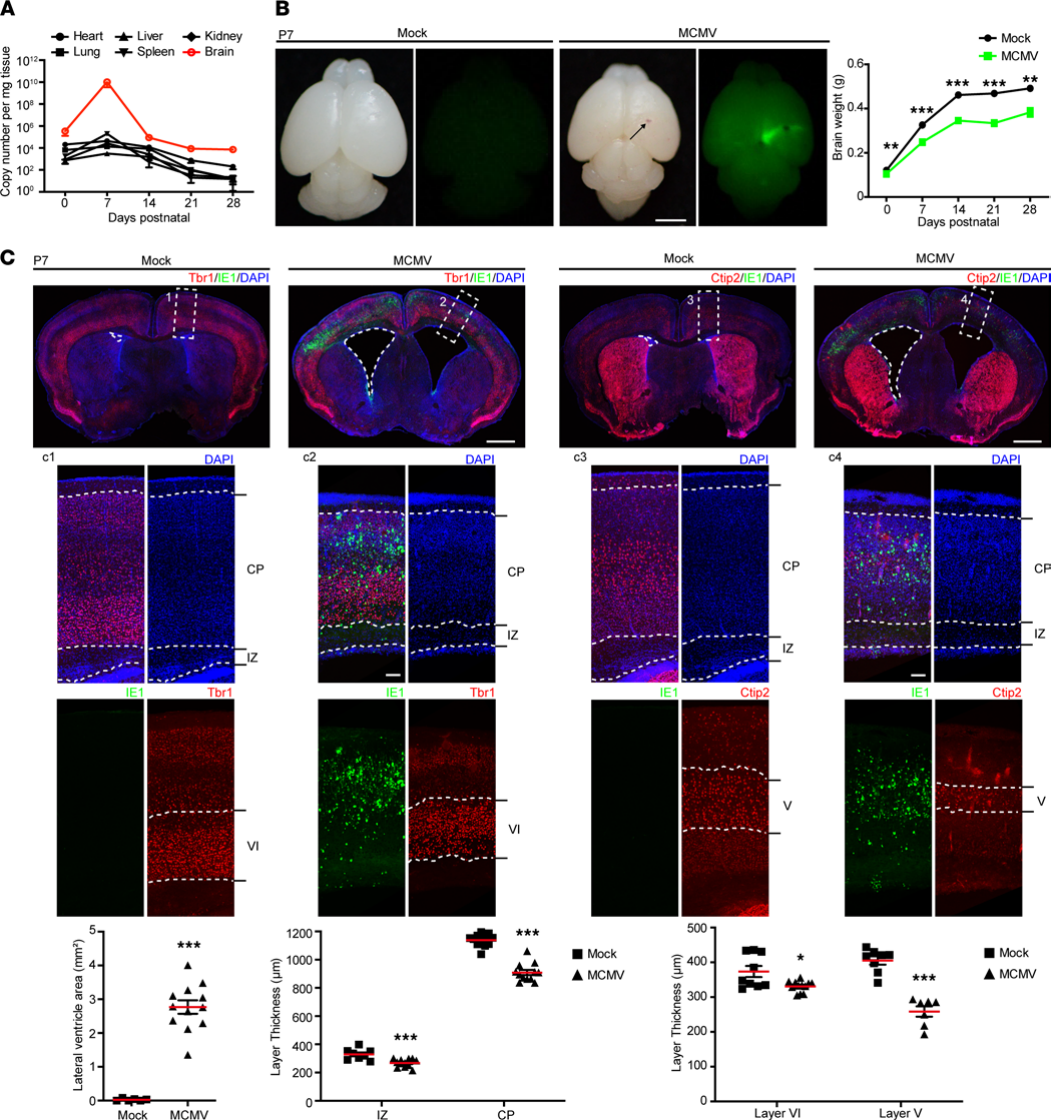
Brain developmental disorders with ventriculomegaly and cortical atrophy. (**A**) Viral loads in different organs. Viral genome copy number was assessed by qPCR using MCMV-DNA extracted from equal amounts of different organ tissues from 3 MCMV-infected mice at the indicated time points. Results are presented as mean ± SEM. (**B**) Dynamic changes in brain weight. Representative images of brains harvested at P7 using bright-field and fluorescence microscopies are shown. Hemorrhage is indicated by black arrow. Brain weights were measured at the indicated time points. Scale bar: 2 mm. (**C**) Lateral ventricle areas and thicknesses of cortical layers at P7. Coronal brain sections were stained for IE1 (green), DAPI (blue), and Tbr1 (red) or Ctip2 (red), respectively. Lateral ventricle areas of position-matched brain sections outlined by white dashed lines were measured. Thicknesses of cortical layers in the position-matched region (indicated in white dashed box) were measured. Data were collected from 3 mice/group in 3 independent experiments. Results are presented as mean ± SEM and analyzed by 2-tailed Student’s *t* test, respectively. *, *P* < 0.05; **, *P* < 0.01, ***, *P* < 0.001. Scale bar: 1 mm or 100 μm in the magnified images.

The observed loss of brain parenchyma at W14 led us to further investigate the pathological and molecular features in the MCMV-infected brain at earlier time points. To identify an optimal observation time point, viral loads represented with MCMV IE1 gene copy number in different organs (heart, lung, liver, spleen, kidney, and brain) were determined at P0, P7, P14, P21, and P28 by quantitative PCR (qPCR). The viral loads peaked at P7 in all the organs with the highest level detected in the brain (Figure 4A). GFP signals generated by the recombinant virus were mainly located in the cortex of the brains at P7 and colocalized with foci of hemorrhage; in addition, brain weights of MCMV-infected mice were significantly lower than those of mock-infected controls at P7, and this difference increased as infection progressed (Figure 4B), consistent with the reduction in the body weight (Figure 1B). These observations were similar to those observed at E18.5 and P5 when high viral dose was used for the initial infection (Supplemental Figure 1). Therefore, a model of cMCMV infection in the fetal brain was established, and P7 was selected as the optimal time point to investigate neuropathogenesis.

We then investigated the MCMV-induced brain structural changes at P7 in greater detail. Compared with the mock control, we found that in MCMV-infected mice: (i) the average area of the lateral ventricles was significantly larger (Figure 4C); and (ii) the cortex was thinner, with thicknesses of intermediate zone (IZ) decreased by 100 μm, cortical plate (CP) decreased by 200 μm, cortex layer VI (layer marker Tbr1) decreased by 50 μm, and cortex layer V (layer marker Ctip2) decreased by 150 μm (Figure 4C). These data demonstrate that cMCMV infection was associated with ventriculomegaly, cortical atrophy, and/or dysgenesis in mice in the early postnatal stage. These findings were consistent with previous reports (22) and the findings in our studies using a high viral dose to initiate infection (Supplemental Figure 2).

### cMCMV infection impairs NPC proliferation and migration in the developing brain.

**Figure 5:**
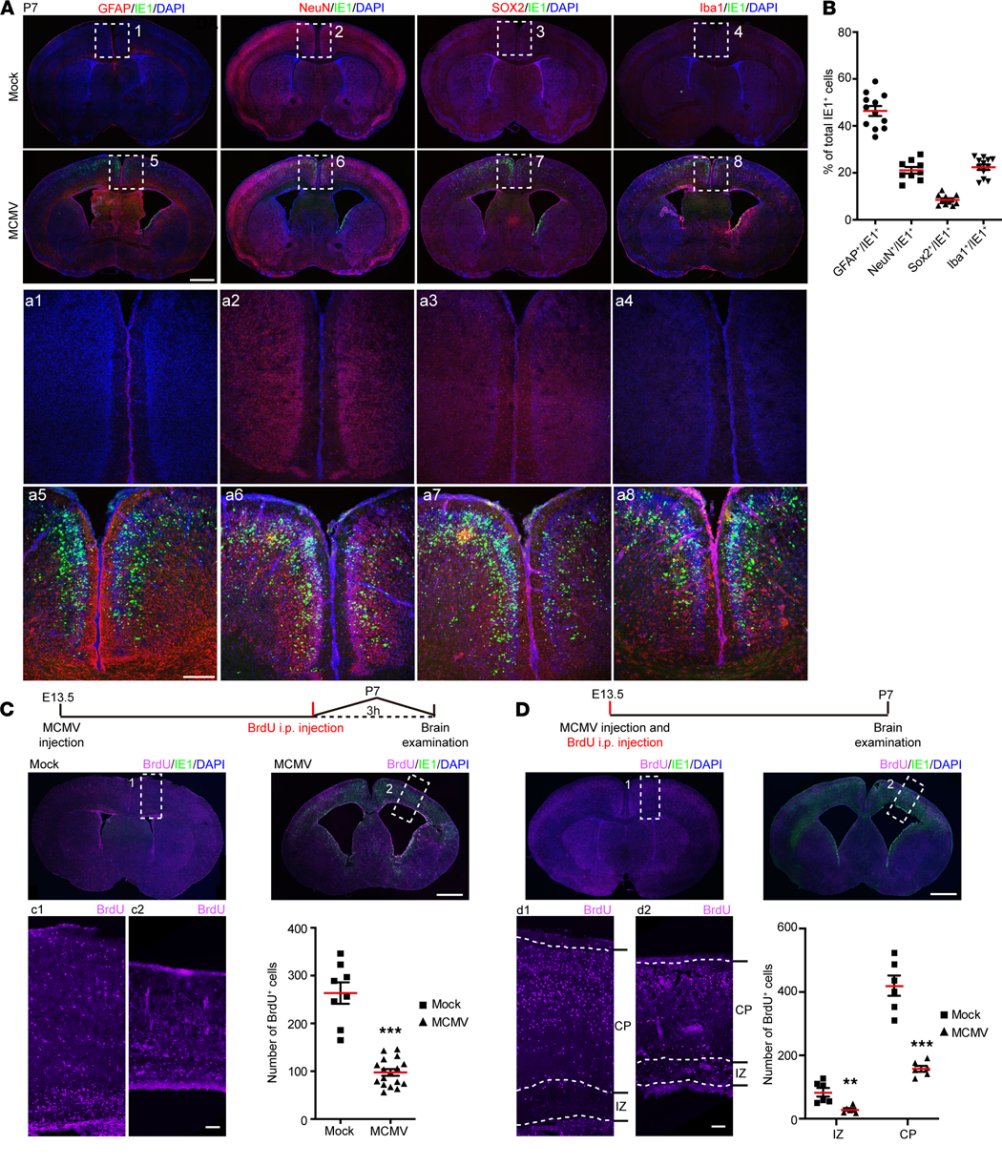
cMCMV infection of brain resident cells and impaired NPC proliferation and migration. (**A**) Localization of IE1 and brain cell markers. Coronal brain sections were stained by immunofluorescence assay (IFA) for IE1 (green), DAPI (blue), or GFAP (red), NeuN (red), SOX2 (red), or Iba1 (red), respectively. Position-matched cortex regions (indicated by white dashed box) are shown in magnified views. (**B**) Percentage of different cell types in IE1^+^ cells. Among the IE1^+^ cells, the proportions of GFAP^+^IE1^+^, NeuN^+^IE1^+^, SOX2^+^IE1^+^, or Iba1^+^IE1^+^ cells in position-matched cortex were quantified separately. (**C**) NPCs’ proliferation at P7. BrdU was administrated i.p. to newborns at P7, and the brains were harvested 3 hours later. Coronal brain sections were stained for BrdU (purple), IE1 (green), or DAPI (blue). BrdU^+^ cells in position-matched cortex (indicated by white dashed rectangle) in each group were quantified. (**D**) NPCs’ migration at P7. BrdU was administrated i.p. to pregnant mice at E13.5, and neonatal brains from offspring were harvested at P7. Coronal brain sections were stained for BrdU (purple), IE1 (green), or DAPI (blue). BrdU^+^ cells in position-matched IZ and CP (indicated by white dashed rectangle) were quantified. Data were collected from 3–9 newborns/group in 3 independent experiments and analyzed by 2-tailed Student’s *t* test. **, *P* <0.01; ***, *P* < 0.001. Scale bar: 1 mm (**A**, top); 200 μm (**A**, bottom); 100 μm (**C** and **D**).

The detection of MCMV IE1 in the cortex of the infected brains at P7 prompted us to investigate which cells in the brain were infected with MCMV. Colocalizations between IE1 and: (i) glial fibrillary acidic protein (GFAP) (an NPC/astrocyte marker), (ii) SRY-box transcription factor 2 (SOX2) (an NPC marker), (iii) NeuN (a neuron marker), and (iv) ionized calcium-binding adapter molecule 1 (Iba1) (a microglia/macrophages marker) in the cortex could be detected at P7 (Figure 5A). About 50% of the IE1^+^ cells were GFAP^+^ (Figure 5B), suggesting that NPCs/astrocytes represented the most common cell type infected in the IZ and CP. Double-positive IE1^+^SOX2^+^ cells were also observed in the ventricular/subventricular zone (VZ/SVZ) (Figure 5A), where NPCs mainly reside. Similarly, most IE1^+^ cells were located around the ventricles and in the VZ/SVZ in the high viral dose model at E18.5 (Supplemental Figure 2, A–C). These data support the previous finding that NPCs are susceptible to cCMV infection in the fetal brain (23). We then assessed the impact of cMCMV infection on NPC proliferation and migration using a BrdU incorporation assay. Compared with the mock control, there were significantly fewer BrdU^+^ cells in the VZ/SVZ of the MCMV-infected brain (Figure 5C). The majority of BrdU^+^ cells migrated to the outer layers of the CP in mock control, while BrdU^+^ cells were most frequently located in the VZ/SVZ and IZ in MCMV-infected brains (Figure 5D). Decreased NPC proliferation and migration were also observed in our high–viral dose model at E18.5 (Supplemental Figure 2, D and E). Together, these data demonstrated that cMCMV infection predominantly targets NPCs and impairs the proliferation and migration of NPCs at various stages of embryonic brain development. This mechanism is consistent with the findings of ventriculomegaly and cortical atrophy and/or dysgenesis in infected mice.

### cMCMV infection induces neuroinflammation with inflammatory cytokine levels and leukocyte infiltration peaking at P7.

**Figure 6:**
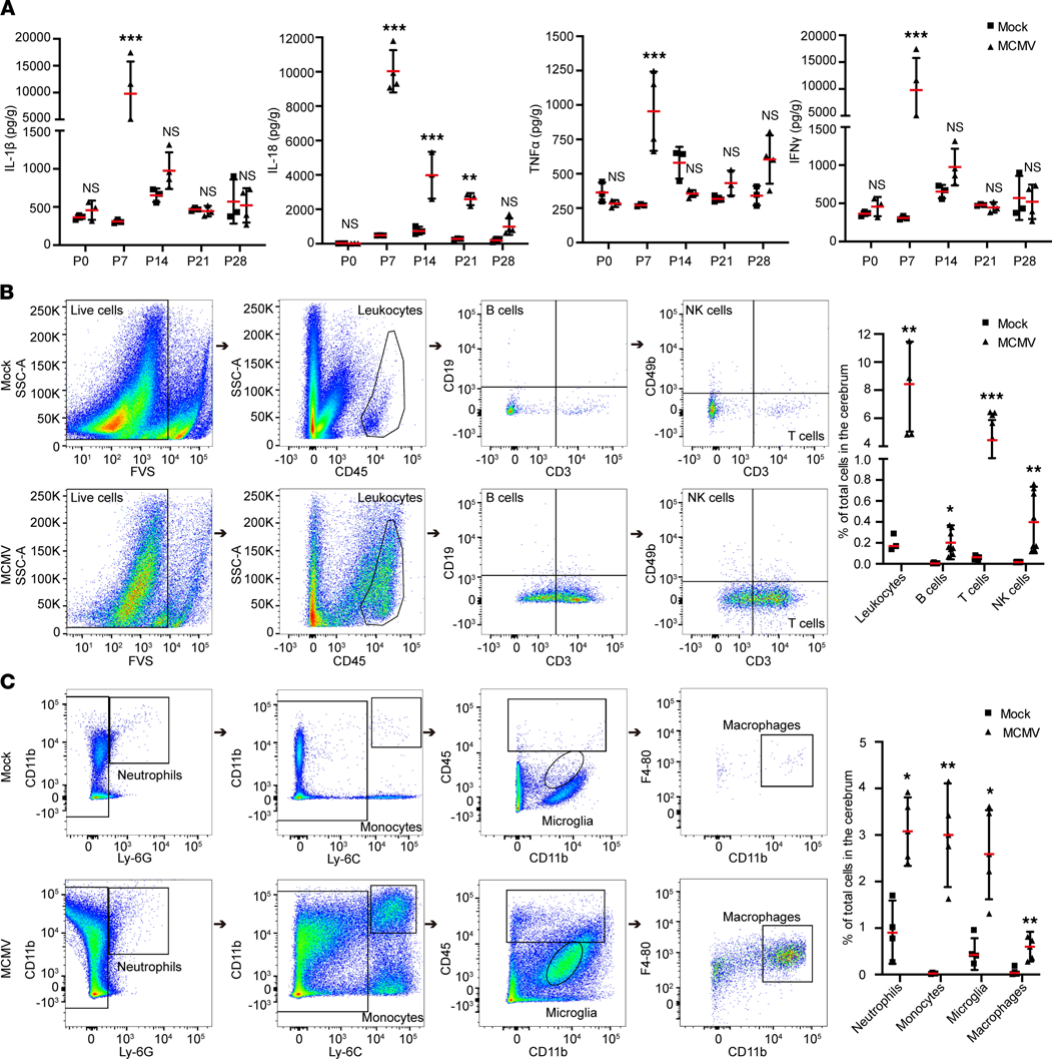
Neuroinflammation induced by cMCMV infection. (**A**) Dynamic changes of cytokine levels in cerebrum of infected mice. Levels of IL-1β, IL-18, TNF-α, and IFN-γ in cerebral samples at the indicated time points were determined by ELISA from 3–6 newborns/group in 3 independent experiments. (**B**) Representative flow cytometric plots of B (CD45^+^CD19^+^), T (CD45^+^CD3^+^), and NK cells (CD45^+^CD3^–^CD49b^+^) among total cells in the cerebrum at P7 are shown. Percentages of leukocytes and B, T, and NK cells in total cells in the cerebrum were calculated from 12 newborns/group. (**C**) Neutrophils, monocytes, microglia, and macrophage infiltration in cerebra at P7. Representative flow cytometric plots separating neutrophils (CD11b^+^Ly-6G^+^), monocytes (CD11b^+^Ly-6C^+^), microglia (CD45^–^CD11b^dim^), and macrophages (CD45^+^CD11b^+^F4-80^+^) among total cells in the cerebrum are shown. Percentages of neutrophils, monocytes, microglia, and macrophages among total cells in the cerebrum were calculated from 12 newborns/group. Data were analyzed by 2-tailed Student’s *t* test and results are presented as means ± SEMs. *P < 0.05; **P < 0.01; ***P < 0.001.

The activation of astrocytes and microglia at P7, indicated by the high-intensity signals in Figure 5A, led us to further characterize the features of neuroinflammation induced by cMCMV infection. The levels of inflammatory cytokines (IL-1β, IL-18, TNF-α, and IFN-γ) in both mock- and MCMV-infected brains were measured by ELISA. Compared with mock control, the levels of each of these cytokines were significantly elevated in MCMV-infected brains, and each peaked at P7 before subsequently declining (Figure 6A). These findings were consistent with a similar peak in viral load at P7 (Figure 3A). Leukocyte infiltration in the cerebrum of infected mice was determined by flow cytometry at P7 at the peak of MCMV-induced neuroinflammation. In flow cytometric analyses, leukocytes were gated by CD45 expression and subdivided based on the expression of CD3, CD19, and CD49b (Figure 6B), while myeloid cells, including microglia, were identified by expression of CD11b, Ly-6G, Ly-6C, and F4-80 (Figure 6C). Compared with mock control, the total number of leukocytes in MCMV-infected brains was increased 40-fold, or about 8% of the total number of cells in the cerebrum. This increase included B cells (CD45^+^CD19^+^), T cells (CD45^+^CD3^+^), and NK cells (CD45^+^CD3^–^CD49b^+^), which respectively represented about 0.2%, 4%, and 0.4% of the total cells in the cerebrum and were 37-, 80-, and 308-fold higher than those in mock control (Figure 6B). Similarly, myeloid cells, neutrophils (CD45^+^CD11b^+^Ly-6G^+^), monocytes (CD45^+^CD11b^+^Ly-6C^+^), microglia (CD45^dim^CD11b^+^), and macrophages (CD45^+^CD11b^+^F4-80^+^) represented, respectively, about 3.1%, 2.9%, 2.4%, and 0.6% of total cells in the cerebrum of MCMV-infected mice and accordingly were 3-, 120-, 5-, and 8-fold higher than those in the mock control (Figure 6C). Taken together, these data demonstrate that cMCMV infection induces a strong neuroinflammatory response and leukocyte infiltration in the brain during the postnatal period.

### cMCMV infection induces cell death in the brain of infected mice via both pyroptosis and apoptosis.

**Figure 7:**
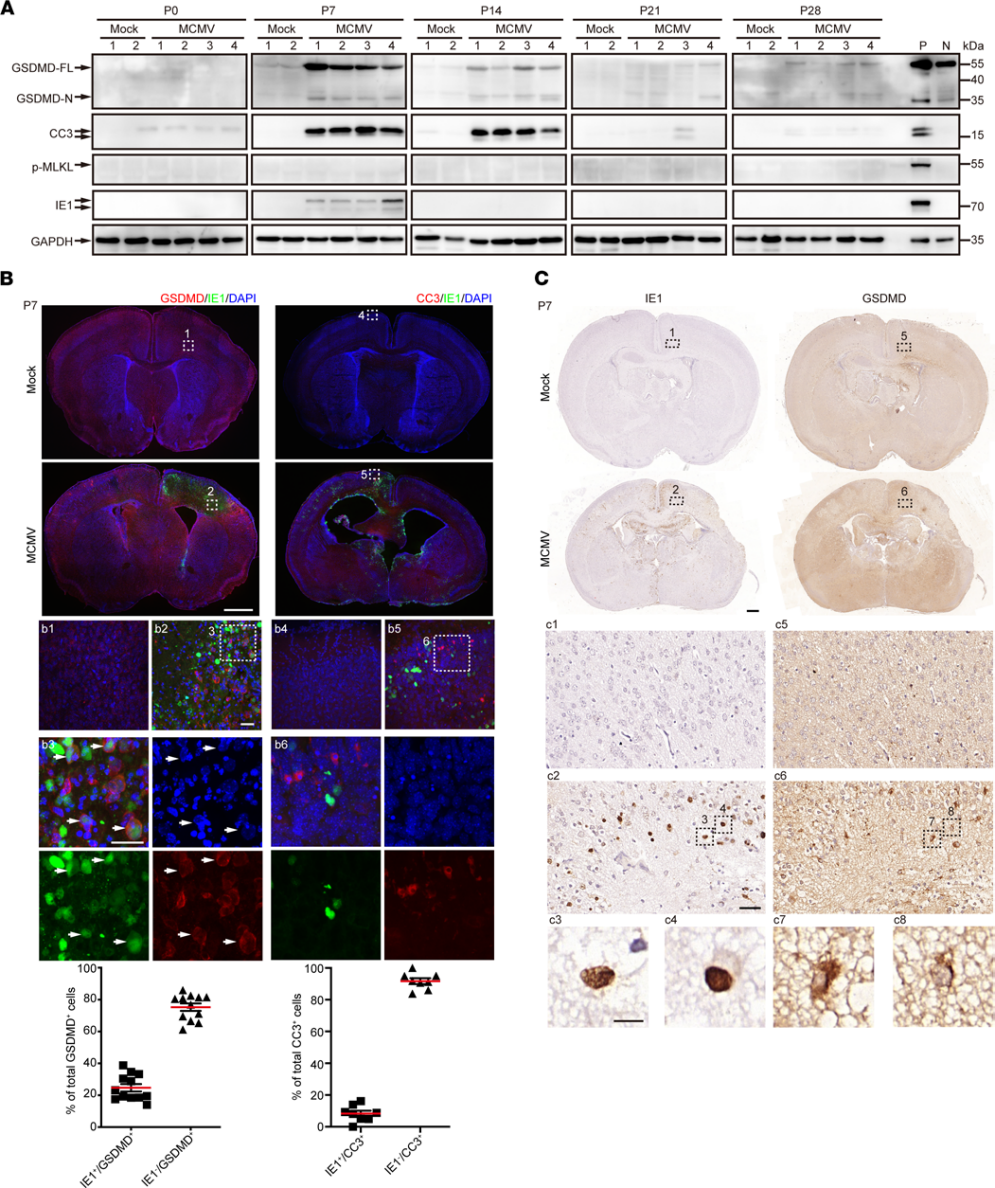
Brain cell death via pyroptosis and apoptosis induced by cMCMV infection. (**A**) Factors in programmed cell death pathways. The factors in pyroptosis (GSDMD-FL and cleaved GSDMD-N), apoptosis (cleaved caspase-3, CC3), necroptosis (p-MLKL), and IE1 in cerebral cell lysates at the indicated time points were determined by Western blot. Positive (P) and negative (N) controls for the target proteins are included to indicate the correct sizes. GAPDH served as a loading control. (**B**) Distributions of GSDMD or CC3 in IE1^+^ cells by IFA. Position-matched coronal cerebral sections of mock- and MCMV-infected brains at P7 were stained for IE1 (green), DAPI (blue), or GSDMD (red) or CC3 (red). Magnified views (indicated by white dashed box) are shown. Colocalizations of GSDMD and IE1 are indicated by white arrowheads. Scale bar: 1 mm (top); 50 μm (bottom). (**C**) Localization of GSDMD in IE1^+^ cells by IHC. Position-matched adjacent sequential coronal cerebral sections of mock- and MCMV-infected brains at P7 were stained for GSDMD and IE. Colocalizations of GSDMD and IE1 are shown in magnified views (indicated by black dashed rectangles). Scale bar: 1 mm (top), 50 μm (middle), 10 μm (bottom).

Regulated cell death is often observed during virus infection and is an important host antiviral response. To investigate cell death pathways in neural cells of MCMV-infected brains, the active forms of a major pyroptosis effector (N-terminus of gasdermin D; GSDMD-N), apoptosis effector (cleaved caspase-3, CC3), or necroptosis effector (phosphorylated mixed lineage kinase domain-like, p-MLKL) were assessed by Western blot. Due to the absence of p-MLKL at all the time points, necroptosis did not seem to be involved in neural cell death. The presence of GSDMD-N and CC3, coinciding with detection of IE1 at P7 (Figure 7A), strongly suggested MCMV-associated activation of pyroptosis and apoptosis, which were also coincident with the peak of IL-1β and IL-18 at P7 (Figure 6A).

We next investigated whether apoptosis and pyroptosis were induced in MCMV-infected neural cells or in uninfected bystander cells. Sequential coronal brain sections at P7 were stained for IE1, CC3, and GSDMD. Most CC3 signals (~90.5%) were present in the cells adjacent to IE1^+^ cells and did not colocalize with IE1 signals, suggesting that mostly bystander cells underwent apoptosis and not the infected cells (Figure 7B). The intense signal from GSDMD localized on the cell membrane, a typical feature of active GSDMD, confirming the activation of pyroptosis. About 25% of GSDMD^+^ cells were IE1^+^ (Figure 7B), suggesting that both infected and uninfected cells underwent pyroptosis. To further validate these phenomena, adjacent sequential coronal brain sections were assessed by immunohistochemistry (IHC). Similarly, GSDMD signals were concentrated at the plasma membrane, showing a typical pyroptotic “ring-of-fire” feature (24), with some signals localized to IE1^+^ cells (Figure 7C). Together, these data indicated that (i) both pyroptosis and apoptosis contributed to neural cell death, (ii) about 25% of the pyroptotic cells were MCMV-infected cells, and (iii) apoptosis was mainly observed in bystander cells and less often in MCMV-infected neural cells.

### cMCMV infection induces abnormalities in CT neuroimaging with brain calcification.

**Figure 8:**
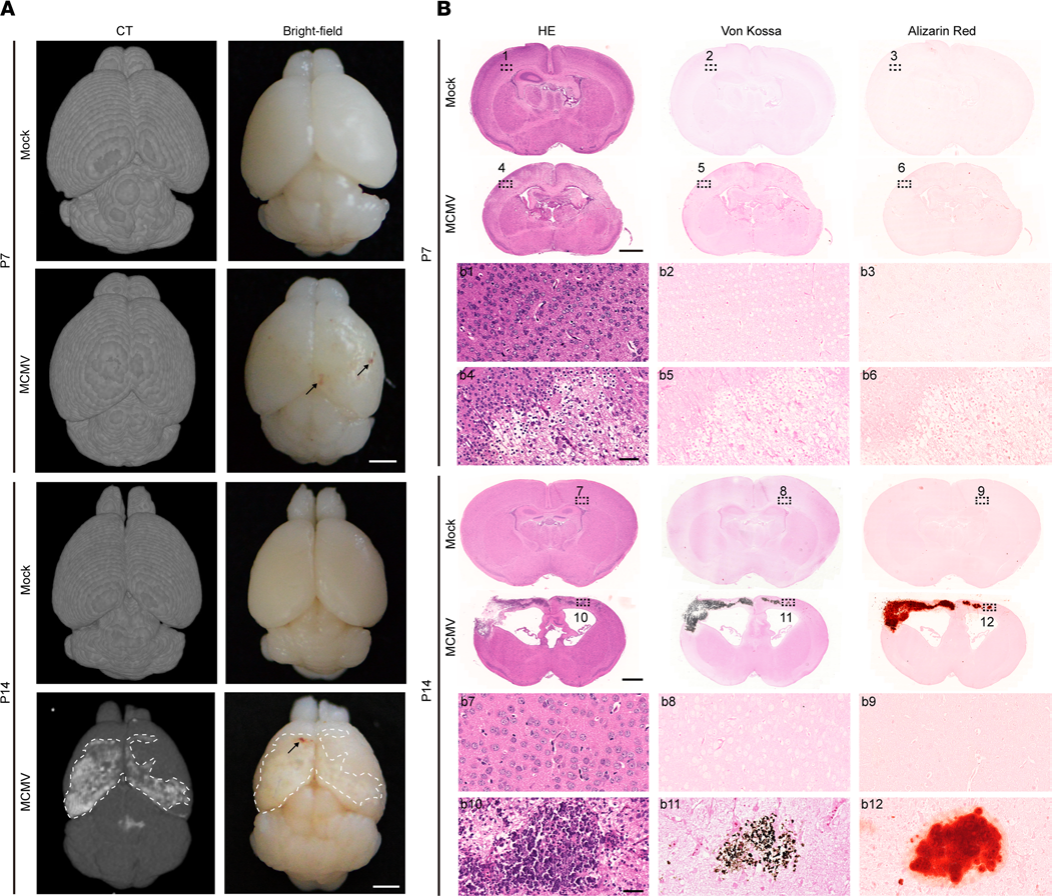
CT neuroimaging abnormalities and brain calcification induced by cMCMV infection. (**A**) Neuroimaging by CT. Neuroimaging by micro-CT was applied to assess brain damage at P7 and P14. Axial views of mock- and MCMV-infected neonatal brains were obtained by bright-field microscopy or micro-CT. Representative images are shown with cerebral calcification area outlined by white dashed lines. Hemorrhage is indicated by black arrows. Scale bar: 2 mm. (**B**) Cerebral calcification assessed by IHC at P7 and P14. Position-matched adjacent sequential coronal cerebral sections of mock- and MCMV-infected brains were subjected to HE staining for brain architecture and von Kossa and Alizarin red staining for calcium deposition. Position-matched regions in the sections are shown in magnified views (indicated by black dashed rectangles). Scale bar: 1 mm; 50 μm (magnified images).

The pathological findings at P7 (brain structure changes, hemorrhage, neuroinflammation, and cell death induced by virus infection) prompted us to further investigate brain damage at later time points. Pathological analyses at P14 revealed pale areas in the cortex and brain lesions with larger areas having evidence of hemorrhage than those observed at P7. By CT neuroimaging, bilateral cortex regions with hyperintense signals were identified in the MCMV-infected brains only at P14 and not P7 (Figure 8A). These areas were examined by histological analyses using adjacent sequential coronal brain sections. Hematoxylin and eosin (HE) staining showed brain structural changes/damages, ventricular enlargement, and intracellular inclusions at P7 that became more apparent at P14. Calcification assessed by both von Kossa and Alizarin red staining showed strong calcification-specific signals associated with pale areas in the cortex (identified by CT) at P14 but not P7, confirming that the hyperintense signals captured by CT were from calcification in the lesions (Figure 8B). These data suggest that brain calcification observed at P14 may be a consequence of MCMV-induced neuroinflammation and neural cell death at earlier time points.

### Antiviral treatment with GCV reduces brain damage and improves neurological function.

**Figure 9:**
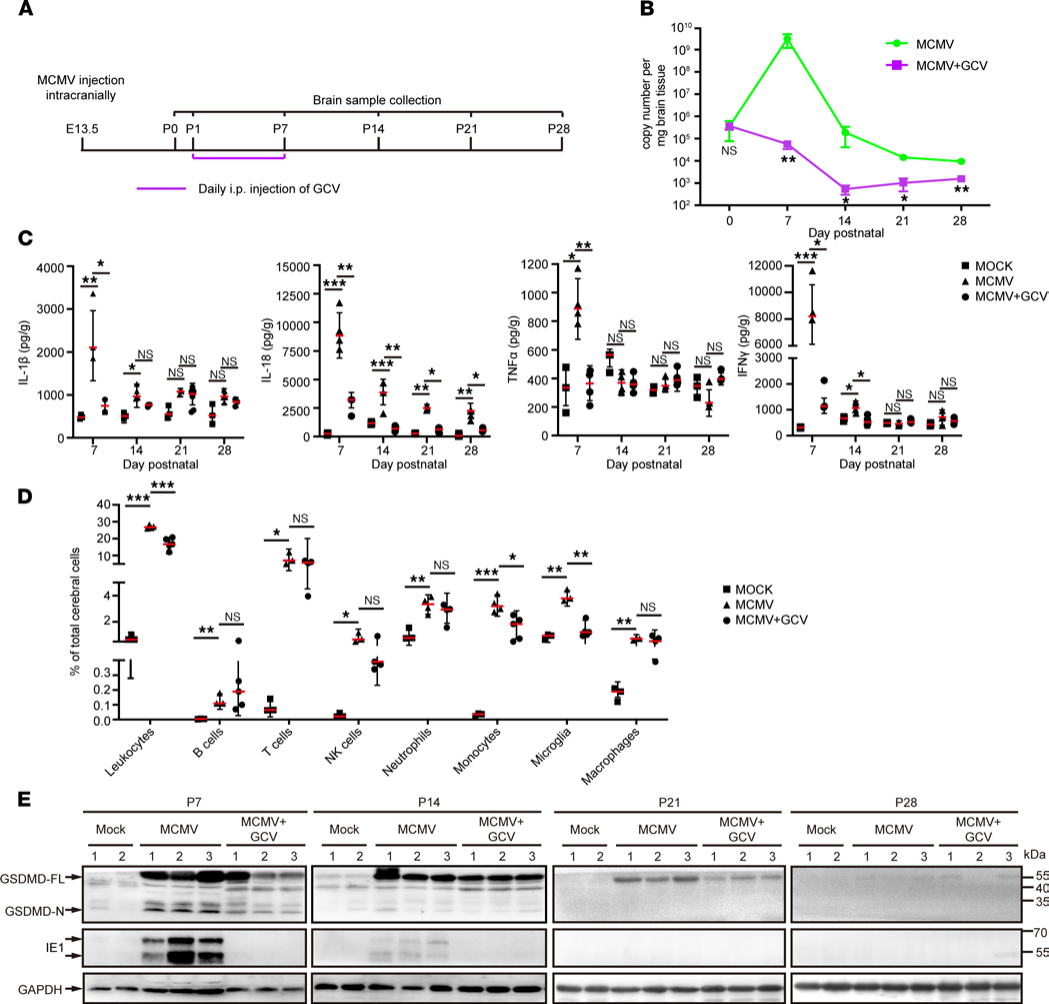
Impact of neonatal GCV treatment on viral load, inflammation, and pyroptosis. (**A**) Timeline of GCV treatment. GCV was injected i.p. daily from P1 to P7. Experimental groups included (i) GCV-treated mock-infected (Mock), (ii) PBS-treated MCMV-infected (MCMV, negative treatment control), and (iii) GCV-treated MCMV-infected (MCMV+GCV) mice. (**B**) GCV treatment and viral loads in brain. Viral genome copy number was assessed by qPCR using DNA extracted from equal amounts of cerebral samples at the indicated time points. Data are from 3 mice/group in 3 independent experiments and analyzed by Student’s *t* test. (**C**) GCV treatment and cytokine levels. Levels of IL-1β, IL-18, TNF-α, and IFN-γ in cerebral samples were determined from 6 newborns/group in 3 independent experiments and analyzed by Student’s *t* test. (**D**) GCV treatment and immune cells infiltration. Percentages of leukocytes; B, T, and NK cells; neutrophils; monocytes; microglia; and macrophages in total cells of the cerebrum from P7 newborns were assessed by flow cytometry. Data were collected from 12 newborns/group and analyzed by Student’s *t* test. (**E**) GCV treatment and pyroptosis. Both full-length and active GSDMD (GSDMD-FL and -N) and IE1 (indicated by arrows) in cerebral lysates were detected by Western blot. GAPDH served as a loading control. For all statistical tests, results are presented as mean ± SEM. *P < 0.05; **P < 0.01; ***P < 0.001.

GCV has been used for the treatment of cCMV infection for decades, including intrauterine treatment of human fetuses infected with HCMV and newborn infants who present at birth with cCMV infections. However, its effect on prevention of HCMV-induced brain damage has not been fully assessed (25, 26). Using our model, we investigated the effects of GCV treatment on CMV-induced, long-term outcomes. GCV was administrated i.p. to mock-infected (mock group) and MCMV-infected newborns (MCMV+GCV group) daily from P1 to P7, with PBS treatment of MCMV-infected newborns serving as a negative control (MCMV group). Brain samples were collected at P0, P7, P14, P21, and P28 (Figure 9A). As expected (27), viral loads in GCV-treated brains were significantly lower compared with the MCMV group, indicating that GCV effectively inhibited viral replication (Figure 9B). The antiviral treatment also significantly improved survival rates (Supplemental Figure 3A) and body weights (Supplemental Figure 3B), and no adverse effects were observed in the mock group given the same GCV treatment (Supplemental Figure 3B). We next investigated MCMV-induced neuroinflammation after antiviral treatment. At P7, levels of IFN-γ, TNF-α, IL-1β, and IL-18 were significantly decreased (Figure 9C), and leukocyte infiltration was also reduced (Figure 9D). Furthermore, both pyroptosis (Figure 9E) and apoptosis (Supplemental Figure 3C) induced by cMCMV infection were decreased. These data indicate that antiviral treatment can effectively control viral replication and consequently alleviate MCMV-induced inflammation and associated cell death.

**Figure 10:**
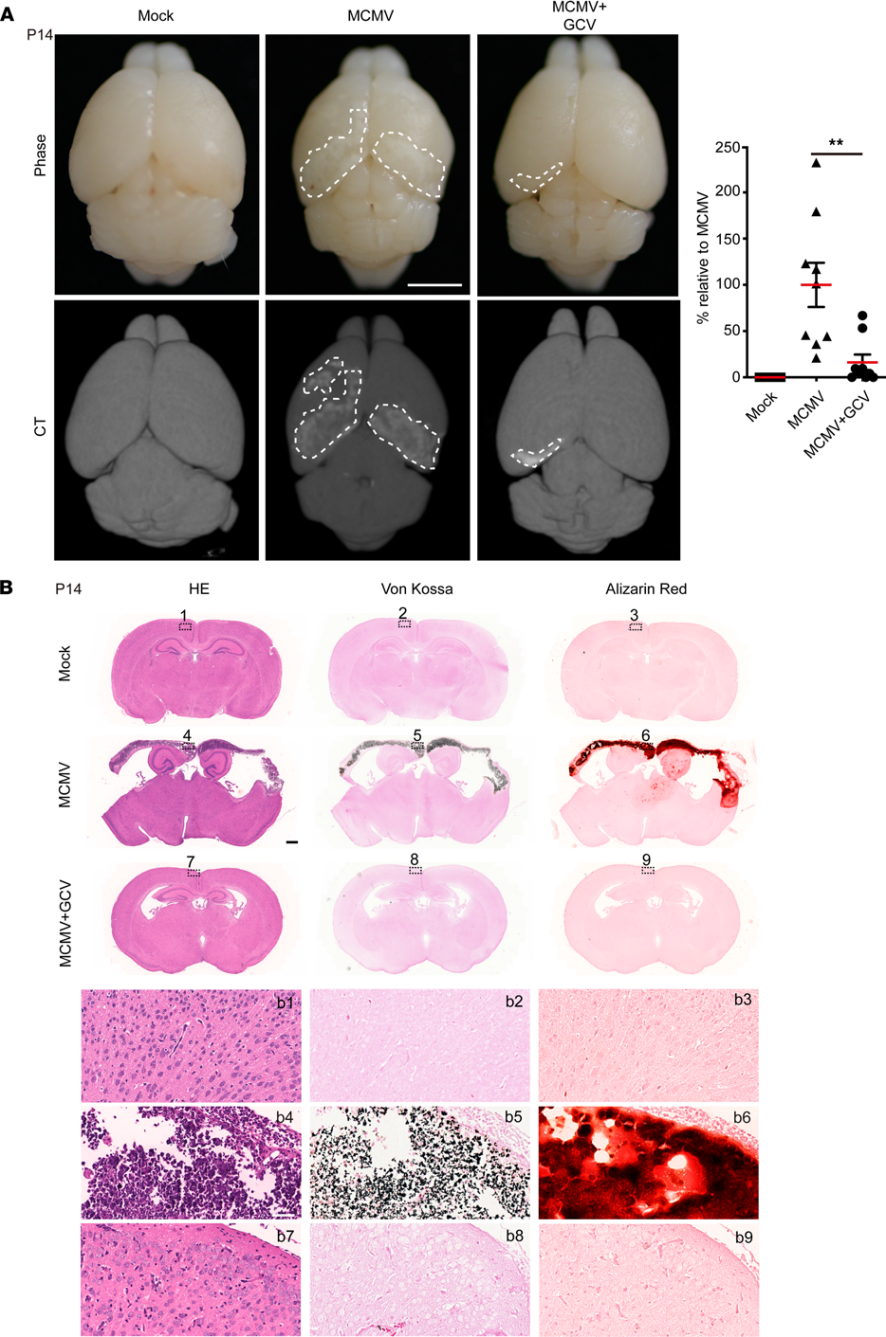
Impact of neonatal GCV treatment on neuroimaging abnormalities and brain calcification. (**A**) Neuroimaging by CT after GCV treatment. Neuroimaging by CT was applied to assess brain damage of mice at P14. Representative CT images with cerebral calcification outlined by white dashed lines are shown. The calcification volume was estimated using imaging software. The percentage of calcification volume relative to that of the MCMV group was calculated. Data were analyzed by 2-tailed Student’s *t* test and results are presented as means ± SEMs. **P < 0.01. Scale bar: 2 mm. (**B**) GCV treatment, tissue loss, and cerebral calcification by IHC. Position-matched adjacent sequential coronal cerebral sections of mice in each group at P14 were stained with HE for brain architecture and with von Kossa and Alizarin red for cerebral calcium deposition. Scale bar: 1 mm; 50 μm (magnified images).

We next investigated if GCV could reduce brain damage, including calcifications, in infected animals. The pale areas and brain lesions identified by CT were noticeably reduced by GCV treatment. Although brain calcification was still observed at P14, the 3D reconstruction of sequential CT images showed that GCV treatment significantly decreased the average calcification volume compared with those of the MCMV group (Figure 10A). Furthermore, pathological analyses confirmed decreased areas and intensities of calcifications in the brains of the MCMV+GCV group when compared with untreated but infected animals at P14. In addition, structural changes in the brain, including the changes in the ventricle area and cortical atrophy, were reduced by GCV treatment, indicating a beneficial effect on brain calcification and brain damage (Figure 10B).

**Figure 11:**
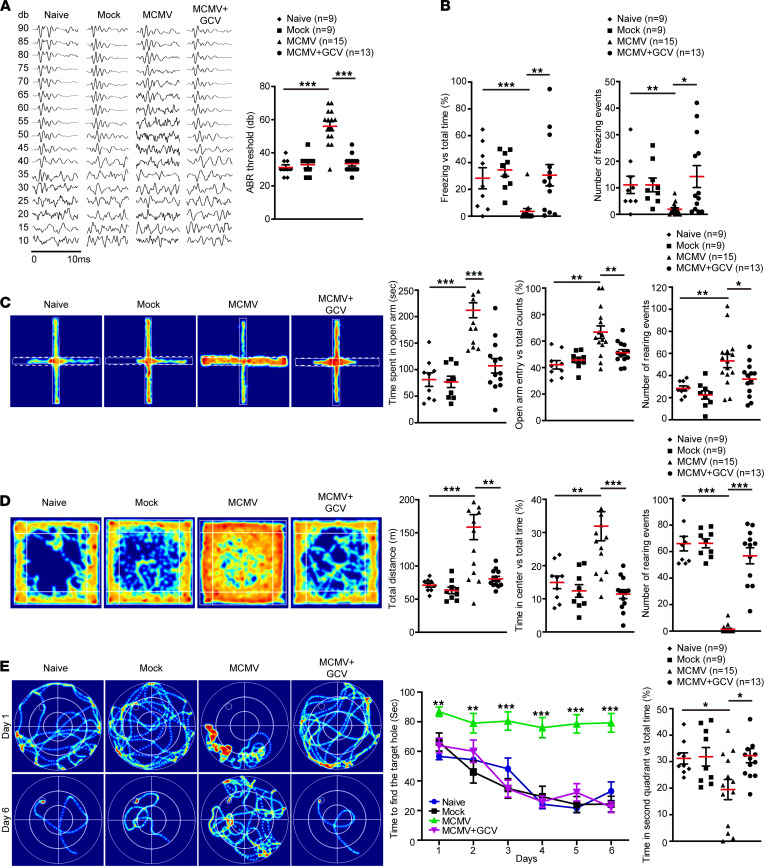
GCV treatment and neurological functions. (**A**) GCV treatment and hearing ability. Hearing ability was determined by ABR test on mice in 4 groups (naive, mock, MCMV, and MCMV+GCV). Examples of 10 ms audiograms of mice from each group over a range of frequencies (clicks) are shown. ABR thresholds were recorded and analyzed by 1-way ANOVA. (**B**) GCV treatment and short-term learning-memory ability. Short-term learning-memory was assessed by fear conditioning test. Proportion of freezing time and number of freezing events were recorded. Data were analyzed by 1-way ANOVA. (**C**) GCV treatment and anxiety-like behavior in EPMT. The general anxiety to open spaces was evaluated by the EPMT. Representative heatmaps of mouse movement tracks and durations are shown. Time spent in the open arms (indicated by white dashed line), percentage of entries to the open arms versus total counts, and number of rearing events of mice were recorded. Data were analyzed by 1-way ANOVA. (**D**) GCV treatment and anxiety-like behavior in OFT. OFT was performed to assess anxiety-like behavior to open area. Representative heatmaps of movement tracks and durations are shown. Total traveled distance, percentage of time traveled in the center, and number of rearing events were measured. Data were analyzed by 1-way ANOVA. (**E**) GCV treatment and spatial learning-memory. Spatial learning-memory abilities were determined by MWM test. Mice were trained in the maze for 6 days and tested on the eighth day. Representative heatmaps of movement tracks and durations are shown. Total time of finding the target platform from day 1 to day 6 and the percentages of time in second quadrant on day 8 were recorded. Data were analyzed by 1-way ANOVA at each time point. For all statistical tests, results are presented as mean ± SEM. *P < 0.05; **P < 0.01; ***P < 0.001.

Finally, we determined if GCV could improve neurological functions, as determined by hearing and behavioral tests. GCV treatment resulted in improved ABR thresholds of mice in the MCMV+GCV group at W7–8 (Figure 11A). Results of behavioral tests were also improved by GCV treatment at W12–14. In cue and contextual fear conditioning tests, mice in the MCMV+GCV group displayed a greater number of freezing events and significantly higher percentages of freezing time over total time than those in the MCMV group (Figure 11B). Similarly, in contrast with the mice in the MCMV group, EPMT showed that mice in the MCMV+GCV group spent significantly less time in the open arm, had smaller percentages of open arm entry over total counts, and had a smaller number of rearing events (Figure 11C). In OFT, mice in the MCMV+GCV group had significantly lower total distances and percentages of time in the center over total time and showed a greater number of rearing events than mice in the MCMV group (Figure 11D). In MWM test, mice in the MCMV+GCV group spent significantly less time finding the target platform and a smaller percentage of time in the second quadrant over total time compared with those in the MCMV group (Figure 11E). Taken together, these improvements in neurological functions are consistent with mitigation of brain damage and calcification resulting from GCV treatment, and importantly, demonstrated that this model is suitable for evaluation of therapeutic strategies targeting neurological defects associated with cCMV infection.

## Discussion

In the current study, we have described an optimized mouse model of congenital infection that infects embryos via i.c. injection with 20 PFU of MCMV at E13.5, resulting in a high survival rate and long survival period that permit neurological testing and imaging. The selection of E13.5 for infection offers the opportunity to study the impact of MCMV infection on the developing brain that reflects infection of the human fetus early in neurodevelopment. Our results demonstrated reproducible brain damage and long-term neurological defects, both of which recapitulate manifestations of cCMV infection during late first and early second trimester (18, 19). In addition to the previously reported ventriculomegaly and cortical atrophy (28), which have been associated with impaired NPC proliferation and migration in the developing brain at embryonic and postnatal stages, our model provides MRI/CT neuroimaging evidence for MCMV-induced brain structural changes and calcifications. Furthermore, pathological and molecular analyses revealed that viral replication and neuroinflammation preceded neural cell death and calcification, implying a causal link between these events. Importantly, GCV treatment mitigated brain damage and improved neurological functions. Taken together, these data demonstrate that the cMCMV infection model will be valuable for investigating CMV neuropathogenesis and evaluating therapeutic strategies for limiting neurodevelopmental sequelae of cCMV infections.

The optimized viral dose, initial infection time, and initial infection site ensure high survival rate and long survival period, facilitating the induction of reproducible long-term neurological defects and brain damage. Mice are considered adult after 6–8 weeks postnatal (W6–8, average W7), and the neurological functions of cognition and learning-memory ability are fully developed after W7 (29). Thus, W7 and later time points are optimal for consistent and reliable neurodevelopment assessments. However, many studies in mouse models of congenital HCMV infections have not performed neurodevelopmental testing beyond W7 (4). Also, models using different infection conditions have resulted in distinct observations, including aberrant brain morphogenesis, neuroinflammation, and inner ear infections (15, 16, 28, 30–32). In some congenital infection mouse models where infection is established by an i.c. injection of 1 × 10^4^ or 1 × 10^5^ PFU of MCMV, infection was only observed in periventricular zones and regions directly connected to cerebrospinal fluid when assayed at E18 (33). Such findings are inconsistent with the widely scattered focal area of parenchymal involvement observed during cCMV infection (34). In another newborn mouse model based on i.p. inoculation of 200 PFU of MCMV, developmental abnormalities were more frequently observed in the cerebellum than in the cerebrum at P7 or P10 (15, 16), and while cerebellar anomalies are common in infants and fetuses with cCMV infection, they are less frequent than cortical abnormalities in some clinical series (35). In this postnatal infection model, cerebellar abnormalities were the most prominent finding of brain maldevelopment, primarily because the cerebellum develops postnatally in rodents (29). In contrast, in our model, CMV infection initiated in the CNS during early neurodevelopment and virus replication continued in the postnatal period, offering an opportunity to observe the potential constellation of neurodevelopmental abnormalities described in clinical descriptions of cCMV.

cCMV infection causes a spectrum of neurological abnormalities, including brain calcification, microcephaly, intellectual disability, SNHL, and seizure, which have not been thoroughly characterized in animal models. Hearing loss has been demonstrated in both infected guinea pig and mouse models (31, 36). A recent study reported i.p. infected newborn guinea pigs developed spatial learning-memory impairments at P15 (8). However, this study lacked an assessment of long-term and systemic neurological defects. In our model, the cMCMV-induced hearing, cognitive, and learning-memory impairments are consistent with neurological defects seen in cCMV-infected children, especially long-term neurological sequelae (12). The observed brain damage occurred often in the cortex area and hippocampus in our model, implying an association between brain structure changes and neurological function as revealed by previous clinical investigations on brain abnormalities and neurological defects (37).

Neuroimaging by MRI and CT are extensively used to aid in the assessment of brain damage in clinical studies of infants with cCMV infections (22). In our model, results demonstrating abnormalities in MRI and CT neuroimaging are described. The abnormalities in brain MRI and CT neuroimaging demonstrate structural damage to the brains of infected mice, which is further confirmed by loss of brain parenchyma in areas matched to abnormal neuroimaging signals. Brain MRI and CT images also demonstrate white matter lesions, ventriculomegaly, and calcifications. These findings are consistent with the frequently observed neuroimaging abnormalities in live newborns with cCMV infection (35). It is notable that the damaged brain areas identified by neuroimaging and pathological analyses, such as the auditory cortex and hippocampus, could be associated with neurological defects that were demonstrated in these mice.

Brain calcification is the most frequent finding associated with cCMV infection that is detected by CT and has been reported to occur in about 50% of the symptomatic congenitally infected children (34, 38). In the cCMV-infected brain, calcification is often found periventricularly, and calcification in the cortex appears as a spot or line (39, 40). Brain calcification detected by CT neuroimaging can be used as a predictor for neurodevelopmental disorders in cCMV-infected children (41). However, brain calcification has seldom been reported in cCMV infection animal models. In our model, the brain calcification is clearly detected by CT neuroimaging, mostly in the cortex and periventricular areas, but the underlying mechanism(s) leading to calcification remain unknown. We have observed sequential events of MCMV infection and inflammation followed by cell death, which appeared to be associated with calcification. Thus, our model provides a platform to investigate the interplay between these events and provide new insights into the mechanisms of calcification.

cMCMV infection results in severe neuroinflammation accompanied by increased cytokine production and infiltration of leukocytes in the brain. Inflammation may contribute to the observed cell death, calcifications, loss of brain parenchyma, and long-term neurological deficits. Neuroinflammation and/or altered immune response and indirect viral cytopathology have been shown in mouse models to be the cause of CMV-induced neurological damage (16, 42). The brain is considered an immune-privileged organ. The inflammatory/immune response induced by pathogens in the brain is normally initiated by the in situ resident innate immune cells such as microglia and astrocytes, followed by leukocytes that infiltrate from the periphery. It has been reported that multiple proinflammatory factors and chemokines such as IFNs, IL-1β, IL-18, and TNF-α are highly induced during CMV infection (43). Although these factors are initially effective in eliminating virus infection in the CNS, high levels can cumulatively be neurotoxic to bystanders surrounding virus-infected cells and eventually lead to neural cell death via pyroptosis and apoptosis. In our model, high levels of inflammatory factors induced by cMCMV infection are consistent with the role of neuroinflammation in cCMV infection-associated brain damage and neurological defects.

Host programmed cell death pathways, including pyroptosis, apoptosis, and necroptosis, are shown to play a role in antiviral activity (44). Previous studies showed that both apoptosis and necroptosis can be activated in CMV-infected macrophages (45). CMVs have evolved several strategies to counteract these cell death pathways and facilitate virus replication. The HCMV UL36 protein and MCMV M36 and M45 proteins are responsible for antagonizing apoptosis and necroptosis to favor viral replication (45, 46), which may explain why fewer MCMV-infected cells exhibit evidence of apoptosis and necroptosis. To date, CMV-induced neuronal cell pyroptosis has not been reported, and the viral factors that trigger or inhibit pyroptosis have not been identified. In our model, activation of pyroptosis and apoptosis but not necroptosis was observed in MCMV-infected brains. The typical ring-of-fire morphology of active GSDMD in MCMV-infected neural cells indicative of GSDMD-mediated pyroptotic cell death was frequently observed. Interestingly, our data show that uninfected cells also undergo pyroptosis and apoptosis in MCMV-infected brains. It will be of great interest to investigate the mechanism of GSDMD-mediated pyroptosis in MCMV-induced neurodevelopmental damage, which may reveal new targets for clinical intervention.

GCV can prevent hearing deterioration in clinical trials (47), and the treatment effects of valganciclovir (a prodrug of GCV) on symptomatic cCMV infection have been evaluated (26). A recent study showed ganciclovir was able to effectively mitigate hearing loss in a murine model of congenital CMV infection (27), and further investigation revealed the role of free radical formation in mediating CMV-induced hearing loss (48). In the current study, the improved neurological functions, reduced brain damage, and improved CT neuroimaging findings with GCV treatment indicate our model could be invaluable for assessing inhibitors for efficacy in protecting the fetus from infection-induced neurological damage. Our model may also serve as a reference for evaluating the optimal strategies for antiviral treatments.

## Methods

### Cell cultures.

NIH3T3, RAW 264.7, and L292 cells were obtained from ATCC. All cells were grown in DMEM supplemented with 10% FBS at 37°C in a 5% CO_2_ incubator.

### Virus, mice, and infection.

MCMV K181-eGFP strain was reconstituted by transfection of bacterial artificial chromosome DNA into NIH3T3 and harvesting of cell culture supernatants 10 days posttransfection as described previously (21). Viral titers were determined in triplicate by plaque-forming assay using NIH3T3 cells as described previously (6). Specific pathogen–free ICR mice were purchased from Beijing Vital River Laboratory Animal Technology (licensed by Charles River Laboratories). ICR mice, 8 weeks old, were mated, and pregnant females, identified by the presence of vaginal plugs at 12 hours, were selected for virus infection. Infection of pups was performed as described previously (49). Briefly, 2 μL of MCMV viral inoculation containing different infectious units of MCMV was injected into the lateral ventricle of fetuses at E13.5, while the same volume of conditioned medium collected from uninfected NIH3T3 cultures was injected in the same manner as mock-infected control. For fetuses harvested at E18.5 by cesarean section, the fetuses in the right sac were MCMV infected, and the fetuses in the left sac were mock infected. For newborns after natural birth, the entire litter from the same mother was either mock or MCMV infected.

### Antiviral treatments.

Mock- and MCMV-infected newborns were injected by injection (i.p.) with GCV (commercial pharmacy) at a dose of 50 mg/kg in 50 μL of sterile PBS. Treatments were administered once a day from P1 to P7. As a negative control, MCMV-infected newborns were i.p. injected with 50 μL sterile PBS alone (vehicle).

### BrdU incorporation assay.

BrdU was used to evaluate cell proliferation and migration as described previously (50). To detect NPC proliferation during embryogenesis, BrdU (100 mg/kg) was administrated by i.p. injection to pregnant mice at E18.5, and then mock- and MCMV-infected fetuses were harvested 3 hours later. To detect NPC proliferation at P7, newborns were injected with BrdU (100 mg/kg) i.p. at P7 and harvested 3 hours later. To detect NPC migration, pregnant mice were injected with BrdU (100 mg/kg) i.p. at E13.5, and fetuses or newborns were harvested at E18.5 or P7, respectively.

### IFA, IHC, and histochemistry.

PBS-perfused brains were fixed in 4% paraformaldehyde, and coronal sections were prepared for IFA (thickness: E18.5, 20 μm; postnatal, 30 μm) or IHC and histochemistry (thickness: 3 μm). Mouse monoclonal antibodies against MCMV IE1 (51); rabbit monoclonal antibodies against SOX2 (Abcam, ab97959), Tbr1 (Abcam, ab31940, ab183032), Ctip2 (Abcam, ab240636), GFAP (Proteintech, 16825-1-AP), Iba1 (Abcam, ab178847), GSDMD (Affinity, AF4012), or CC3 (Cell Signaling Technology, 9661); and rat monoclonal antibodies against BrdU (Abcam, ab6326) or Ctip2 (Abcam, ab18465) were used in IFA or IHC as indicated. Secondary antibodies for IFA included Alexa Fluor 488 goat anti–mouse IgG1 (Invitrogen, A-21121), Alexa Fluor 568 donkey anti–rabbit IgG (H+L) (Invitrogen, A-10042), Alexa Fluor 647 goat anti–mouse IgG1 (Invitrogen, A-21240), and Alexa Fluor 647 goat anti–rat IgG (H+L) (Invitrogen, A-21247). DAPI (Life Technologies) was used for nuclei counterstaining. Horseradish peroxidase–conjugated antibody (Proteintech, KIHC-5) was used for IHC. IFA, IHC, and histochemistry were performed as described previously (52).

### qPCR.

For viral load determination, DNA was extracted from heart, lung, liver, spleen, kidney, brain, and placenta of MCMV-infected mice at P0, P7, P14, P21, and P28 using a total genomic DNA extraction kit (Tiangen) according to the manufacturer’s instructions. MCMV IE1 gene copy numbers were determined by qPCR using a CFX-96 Connect system (Bio-Rad) with an iQ SYBR Green Supermix kit (Bio-Rad) using the primers specific to IE1 coding sequences (forward primer sequence: 5′-AGCCACCAACATTGACCACGCAC-3′, reverse primer sequence: 5′-GCCCCAACCAGGACACACAACTC-3′). Each sample was tested in triplicate for 3 independent experiments. Viral load was presented as the mean value of IE1 copy number per milligram of tissue with SDs.

### ABR.

ABR testing was performed with an electrophysiology platform (Tucker-Davis Technologies, RRID: SCR_006495). Needle electrodes were placed subcutaneously (vertex, bilateral ears) on anesthetized W7–8 mice with 0.7% pentobarbital sodium (10 mL/kg, i.p.). Acoustic stimuli (clicks) generated with TDT System 3 (RP 2.1, PA 5, ED 1, Tucker-Davis Technologies) were presented at the rate of 10 per second from 90 dB to 10 dB sound pressure level in a descending sequence of 5 dB steps until no discernible waveform was acquired. Resistance between each electrode and the ground electrode was less than 1 kΩ. ABR signals were band-pass filtered (300 Hz high-pass, 3 kHz low-pass, 0 Hz notch), averaged, and digitized with a TDT RZ6 multifunctional processor controlled by BioSigRP software (Tucker-Davis Technologies). ABR thresholds were defined as the lowest intensity signal at which an ABR waveform was detectable.

### Cued and contextual fear conditioning.

Cued and contextual fear conditioning tests were performed through an unconditioned stimulus to connect the shock to the entire environment. Mice at W12–13 were individually placed into the closed operation box equipped with electrical stimulation and allowed to adapt to the environment for the first 3 minutes followed by 3 times of electrical stimulation for 30 seconds. After 24 hours the mice were put into the same closed operation box for 5 minutes without any electrical stimulations. The animal’s unconditioned fear was presented as the mean value of freezing times and duration of the mice recorded in 3 independent experiments with SDs (53).

### OFT.

W12–13 mice were transported from the holding facility to the testing room in their home cages and left there undisturbed for at least 30 minutes. Each animal was placed in a 60 × 60 × 25 cm^3^ open field chamber and tested for 10 minutes. Mice were monitored throughout each test session by video tracking and analyzed using MATLAB (MathWorks). Mice were placed individually into the center (40 × 40 cm^2^) of the open field arena and allowed to explore freely. The overall motor activity was quantified as the total distance traveled. Anxiety was quantified by measuring the number of rearing events and the percentage of the time and distance spent in the center versus periphery of the open field chamber as described previously (53).

### EPMT.

W12–13 mice were transported as above and left undisturbed for at least 1 hour. A maze made of opaque plastic consisted of 4 arms, each 5 cm wide and 30 cm long, elevated 50 cm above the floor. Two arms were enclosed by 15 cm high walls, and the other 2 were open. Each mouse was placed in the center of the maze and allowed to explore the apparatus for 5 minutes. The number of rearing events, percentage of entries to open arms to total counts, and time spent in the open arms were assessed as described previously (54).

### MWM.

The maze used was a 1.5 m diameter circular pool containing water maintained at 23°C and rendered opaque by addition of nontoxic white paint, as described previously (53). W13 mice were trained for 6 days to find a 10 cm diameter platform hidden at a fixed position beneath the water’s surface. Each daily trial block consisted of 4 swimming trials (15-minute interval between trials). Mice were released at 3 different randomized release points of the pool. The mice rested for 1 day after the 6-day training session, then performed a 1-probe trial without the platform on day 8. In both training and probe trials, the time to find the platform and time spent in the area where the platform was previously located were recorded and presented as mean values with SD.

### MRI neuroimaging.

MRI experiments of W8–9 mice were conducted using a 7.0 T animal MRI scanner (Bruker BioSpec 70/20 USR) using a commercial 72 mm diameter transmit-only birdcage coil and a 20 mm diameter receive-only surface coil. Mice were preanesthetized using 3% isoflurane, and anesthesia was maintained during the MRI scan with 1%–1.5% isoflurane. Respiration rate was monitored continuously. T2-weighted images of brains were obtained using rapid acquisition with relaxation enhancement sequence with the following parameters: repetition time = 3000 ms; echo time= 36 ms; number of average = 4; matrix = 256 × 256 × 24; field of view = 20 × 20 × 9.6 mm^3^; voxel size = 78 × 78 × 40 μm^3^.

### CT neuroimaging.

Micro-CT scanning was performed on PBS-perfused, 4% paraformaldehyde–fixed whole brains collected at P7 and P14. Scanning was conducted with a high-resolution micro-CT system (Bruker Skyscan 1176) with the following scan parameters: tube voltage = 50 kV; tube current = 500 mA; exposure time = 275 ms; rotation step = 0.7°; covered angle = 180°; voxel size = 35 × 35 × 35 μm^3^; filter, 0.5 mm aluminum. Image reconstruction and data processing and analyses were performed using the commercial software provided by the manufacturer. Whole brain masks were obtained by thresholding segmentation of the reconstructed images then intersecting with the original reconstructed image. Another threshold segmentation was then performed to separate calcified tissue from the normal brain tissue to calculate the volume of both calcified areas and whole brain.

### Flow cytometry.

PBS-perfused mouse cerebra at P7 were digested with a Neural Tissue Dissociation Kit (Miltenyi Biotec), and cell debris and residual red blood cells were removed using the Debris Removal Solution (Miltenyi Biotec) and Red Blood Cell Lysis Solution (Miltenyi Biotec) according to the manufacturer’s instructions, respectively. The cells were stained with antibodies against mouse antigens of CD45 APC-Cy7 (BD Biosciences, 557659), CD4 PerCP (BD Biosciences, 553052), CD8a BV650 (BD Biosciences, 563234), CD3 MolCpx BV421 (BD Biosciences, 564008), CD19 PE-CF594 (BD Biosciences, 562291), CD49b APC (BD Biosciences, 560628), Ly-6G BUV395 (BD Biosciences, 565964), Ly-6C PE-CF594 (BD Biosciences, 562728), CD16/CD32 PE-Cy7 (BD Biosciences, 560829), F4/80 Alexa Fluor 647 (BD Biosciences, 565853), or CD11b BV421 (BD Biosciences, 562605) at room temperature for 20 minutes, then washed twice with Cell Staining Buffer (BD Biosciences, 554657). The stained cells were resuspended with 300 μL of Cell Staining Buffer and applied to a BD LSRFortessa Flow Cytometer (BD Biosciences).

### ELISA.

Cerebra samples isolated from PBS-perfused mock- and MCMV-infected brains were minced in PBS supplemented with protease inhibitors (Roche), then centrifuged at 5000*g* for 10 minutes, and supernatants were assayed for TNF-α (Absin), IFN-γ (Absin), IL-1β (Absin), or IL-18 (R&D Systems) following the manufacturer’s instructions.

### Western blot.

Cerebral protein samples were prepared from PBS-perfused brains minced with RIPA buffer supplemented with protease inhibitors as described previously (49). Proteins were separated by SDS-PAGE, transferred onto PVDF membranes, and probed with anti-GSDMD (Cell Signaling Technology, 39754; Abcam), anti–cleaved caspase-3 (Cell Signaling Technology, 9661), anti–p-MLKL (Cell Signaling Technology, 37333), anti-IE1 (51), or anti-GAPDH (MilliporeSigma, G9545) antibodies followed by corresponding peroxidase-conjugated secondary antibodies (MilliporeSigma, AP106P and AP160P). RAW 264.7 cells either treated with LPS (200 ng/mL) followed by Nigericin (10 μM) (positive control) or treated with DMSO (negative control) were used to indicate the correct sizes of GSDMD-FL and GSDMD-N as described previously (55). NIH3T3 cells treated with TNF-α (50 ng/mL) (positive control) or without (negative control) were used to indicate the correct size of CC3. L929 cells treated with TNF-α (50 ng/mL) in the presence of cycloheximide (250 ng/mL) and zVAD (50 μM) (positive control) or with DMSO (negative control) were used to indicate the correct size of p-MLKL as described previously (45). MCMV-infected (positive control) or mock-infected (negative control) NIH3T3 cells were used to indicate the correct size of IE1 (51). See complete unedited blots in the supplemental material.

### Statistics.

A χ^2^ test was used to compare proportions of double-positive cells for immunofluorescence studies. Numerical results were expressed as the mean ± SEM obtained from at least 3 independent experiments. Statistical analyses were performed using unpaired 2-tailed Student’s *t* test, 1-way ANOVA, or Mantel-Cox test. *P* values less than 0.05 were considered significant. The GraphPad Prism 5 software package (GraphPad) was used to calculate *P* values, and the following convention was used: *P ≤ 0.05; **P ≤ 0.01; ***P ≤ 0.001.

### Study approval.

All mouse experiments were approved by the Ethics Committee on Animal Experiments at Wuhan Institute of Virology Institutional China (WIVA10201504) and were performed in accordance with the *Guide for the Care and Use of Laboratory Animals* as published by the US NIH (National Academies Press, 2011). All efforts were made to minimize pain and suffering and the number of animals used.

## Author contributions

MHL, XJ, and YPZ conceived and designed the research. YPZ, MJM, and XJ performed the experiments. XZW, SNH, HBQ, XD, SC, LW, BY, XFA, WBZ, XJL, and HC provided advice and technical assistance. LC provided technical support for ABR testing. XYL, ADH, BZ, and YL provided technical support for neurological testing. MZ, HCL, HL, and XZ provided technical support for CT and MRI detection. QT provided antibodies against viral proteins and technical support for IFA and Western blot. AJR provided MCMV K181 strain. WGZ provided technical support for histological assays. SR, QT, MAM, and WJB provided guidance on experimental design and manuscript revision.

## Supplementary Material

Supplemental data

## Figures and Tables

**Table 1:**
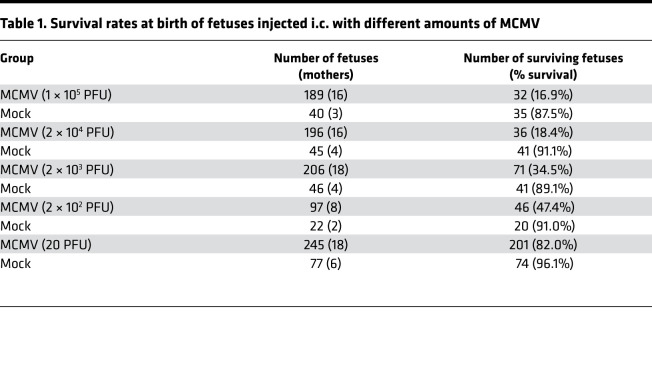
Survival rates at birth of fetuses injected i.c. with different amounts of MCMV

## Version 1 01/11/2022

Electronic publication

## Version 2 04/19/2022

Authors' affiliations were updated.
