# Supplementary material for: A congenital CMV infection model for follow-up studies of neurodevelopmental disorders, neuroimaging abnormalities, and treatment
Source: JCI Insight. 2022 Jan 11;7(1):e152551. doi: 10.1172/jci.insight.152551 (PMC8765053; doi:10.1172/jci.insight.152551)

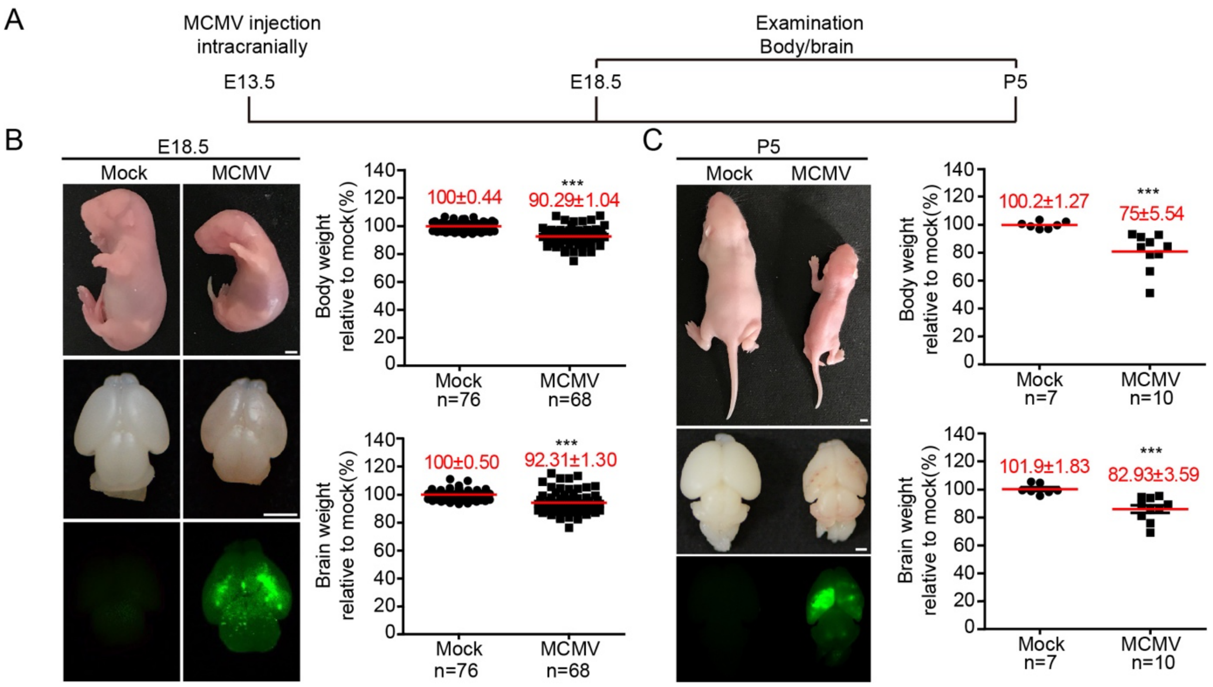

Supplemental Figure 1. cMCMV infection induced developmental delay. **(A)** Timeline of a cMCMV infection mouse model and examinations. A dose of 1×10^5 pfu MCMV (MCMV) or equivalent volume of conditional medium (Mock) was injected intracranially at embryonic day 13.5 (E13.5). General growth, body and brain of the fetuses/newborns were examined at E18.5 and postnatal day 5 (P5). Growth and brain development at E18.5 **(B)** and P5 **(C)**. Representative light field and fluorescence dark field images of whole mouse and brains are shown. Body and brain weights were measured, calculated as percentages of MCMV to mock and analyzed by student's t-test. Results are presented as mean ± SEM. Sample size n is shown for each group. ***, $p < 0.001$. Scale bar: 2 mm.

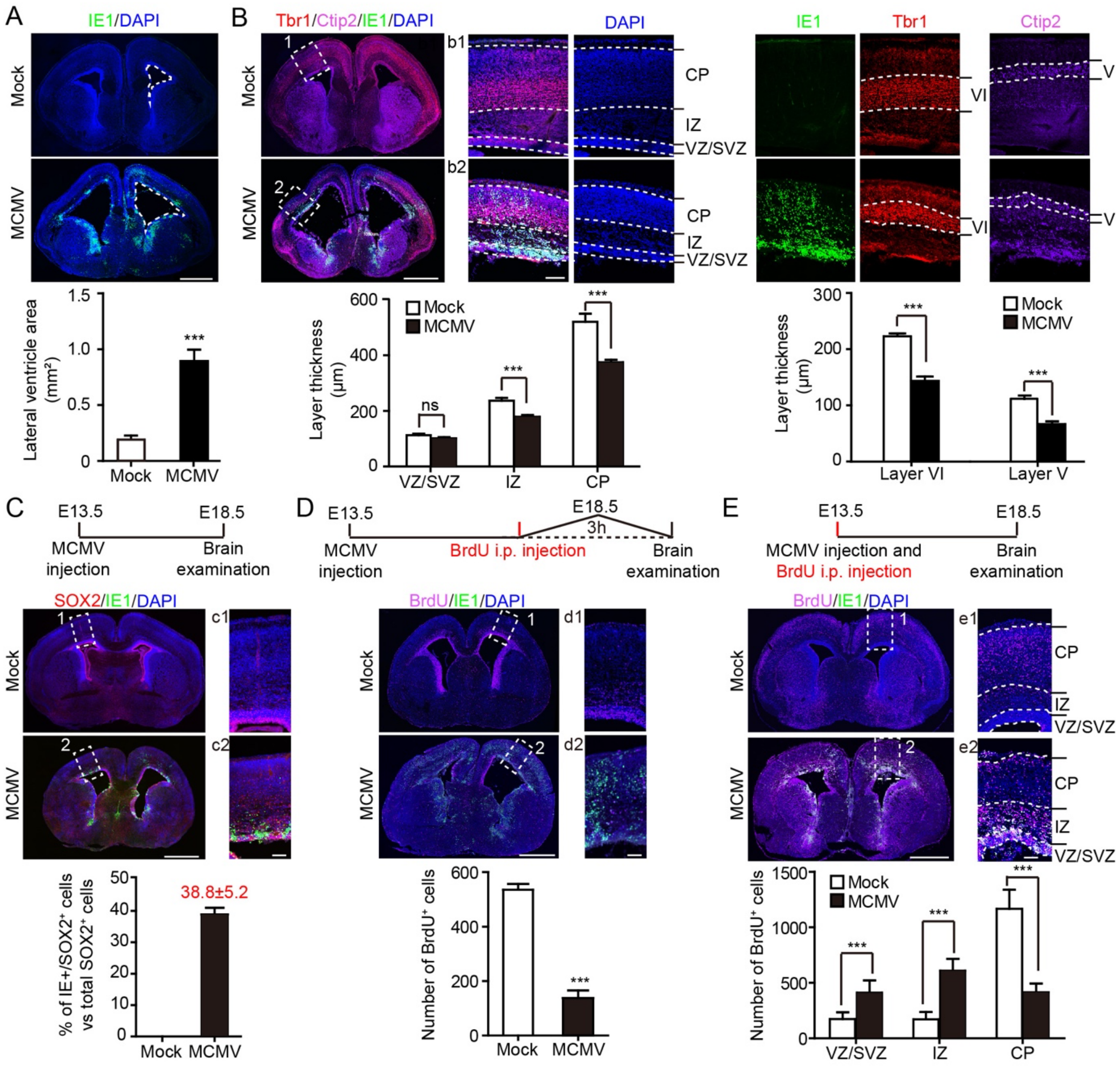

Supplemental Figure 2. cMCMV infection induced fetal brain ventriculomegaly and cortical atrophy, and NPCs proliferation and migration impairment. (A) Lateral ventricle areas at E18.5. Coronal brain sections were stained for IE1 (green) and DAPI (blue). Lateral ventricle areas (outlined by white dashed line) of position-matched mock- and MCMV-infected brain sections were measured. **(B)** Thicknesses of cortical layers at E18.5. Coronal brain sections were stained for Tbr1 (layer IV marker, red), Ctip2 (layer V marker, purple), IE1 (green) and DAPI (blue). The thicknesses of the cortical layers in the position-matched region (indicated by white dashed-line rectangle) were measured. **(C)** Fetal brain NPCs, the main target for congenital MCMV infection. Coronal brain sections of mock and MCMV at E18.5 were stained for NPC marker SOX2 (red), IE1 (green), and

DAPI (blue). Among the SOX2^{+} cells, the percentage of IE1^{+} cells in the position-matched region (indicated by white dashed-line box) was quantified. **(D)** Proliferation of fetal brain NPCs. BrdU was intraperitoneally (i.p.) injected to the pregnant mice at E18.5, and fetal brains were harvested 3 hours (3h) later (indicated in the timeline). Coronal sections of fetal brains were stained for BrdU (purple), IE1 (green), and DAPI (blue). BrdU^{+} cells in position-matched region (indicated by white dashed-line rectangle) were quantified. **(E)** Migration of fetal brain NPCs. BrdU was i.p. injected to pregnant mice at E13.5, and fetal brains were harvested at E18.5 (indicated in the timeline). Coronal sections of fetal brains were stained for BrdU (purple), IE1 (green), and DAPI (blue). BrdU^{+} cells in VZ/SVZ, IZ and CP in position-matched region (indicated by white dashed-line box) were quantified. All data were collected from 3 mock and 9 MCMV fetuses/group in 3 independent experiments and analyzed by student's t-test. Results are presented as mean ± SEM. ns, not significant; ***, $p < 0.001$. Scale bar: 1 mm; or 100 μm in the amplified image.

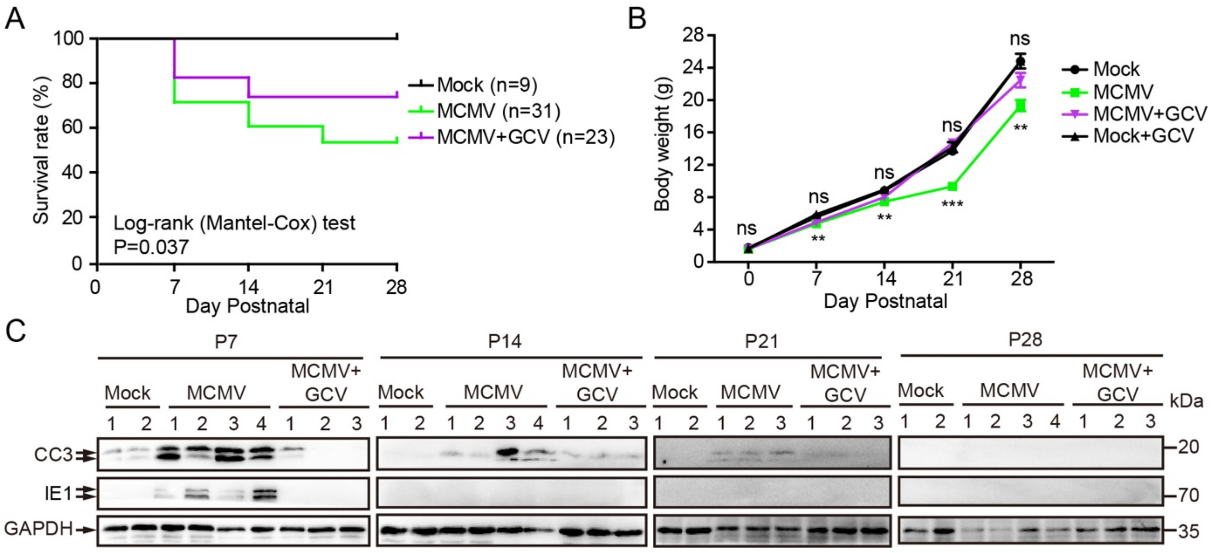

Supplemental Figure 3. GCV treatment: improved survival rates and body weights, and inhibited apoptosis. (A) GCV treatment and change in survival rates. The survival rates of newborns were assessed during P0-28 in the following three groups: GCV-treated mock-infected (Mock), PBS-treated MCMV-infected (MCMV), GCV-treated MCMV-infected (MCMV+GCV) mice. Data were collected from 3-9 newborns/group in 3 independent experiments, and analyzed by Mantel-Cox test. Sample size n of each group is indicated. **(B)** GCV treatment and change of body weights. Body weights of newborns in the Mock, MCMV, GCV-treated mock (Mock+GCV) and MCMV+GCV groups were assessed during P0-P28, 3-9 newborns/group in 3 independent experiments, and analyzed by one-way ANOVA test. Results are presented as mean ± SEM. $^{**}p<0.01$; $^{***}p< 0.001$. **(D)** GCV treatment and apoptosis factor. CC3 and IE1 in cerebral cell lysate samples of newborns in the three groups at the indicated time points were detected (indicated by arrows) by western blot. GAPDH served as a loading control.

Figure 7A
P0

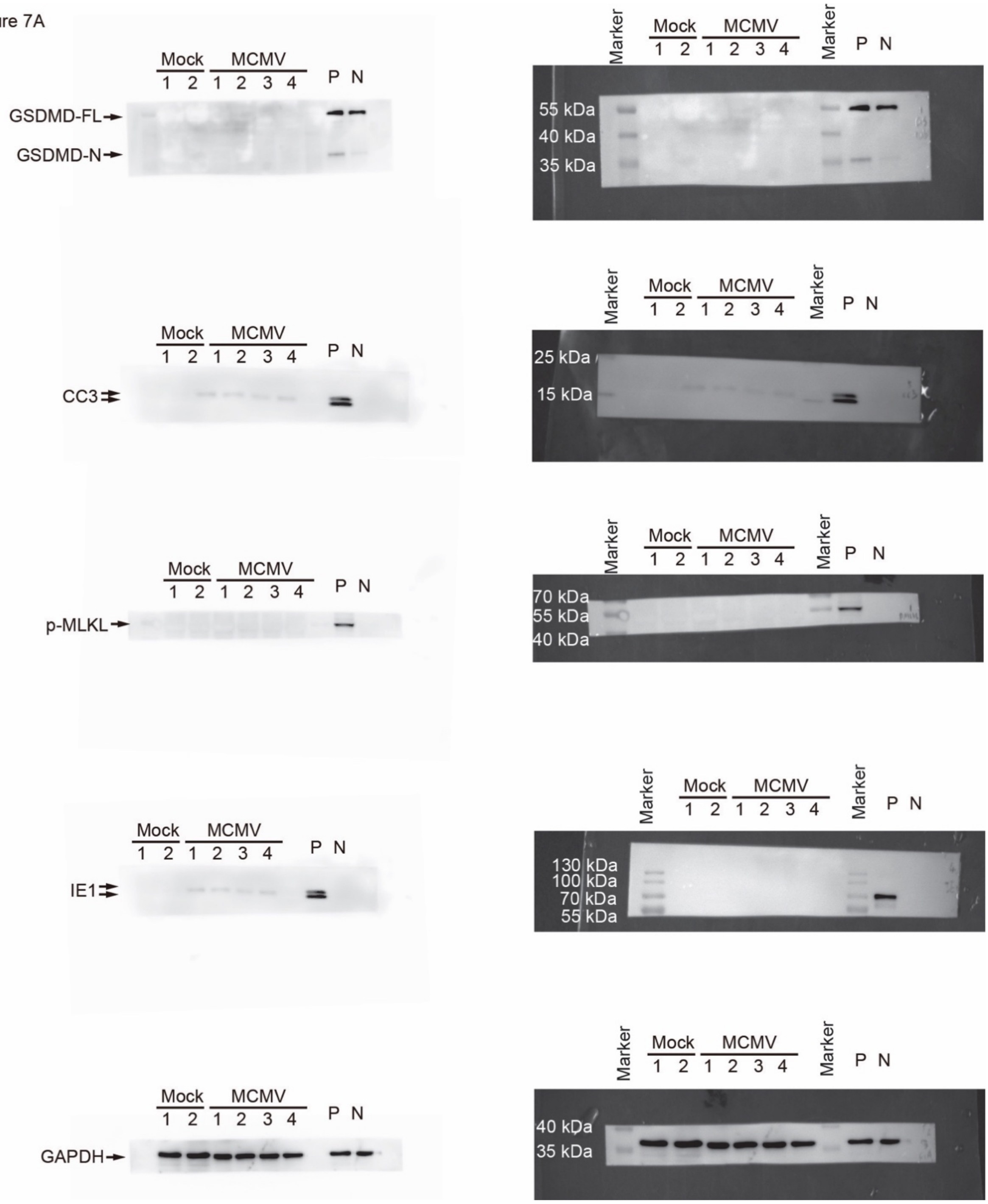

Mock
MCMV
1 2 1 2 3 4
P N
GSDMD-FL
GSDMD-N
CC3
p-MLKL
IE1
GAPDH
Marker
55 kDa
40 kDa
35 kDa
25 kDa
15 kDa
70 kDa
130 kDa
100 kDa

Figure 7A
P7

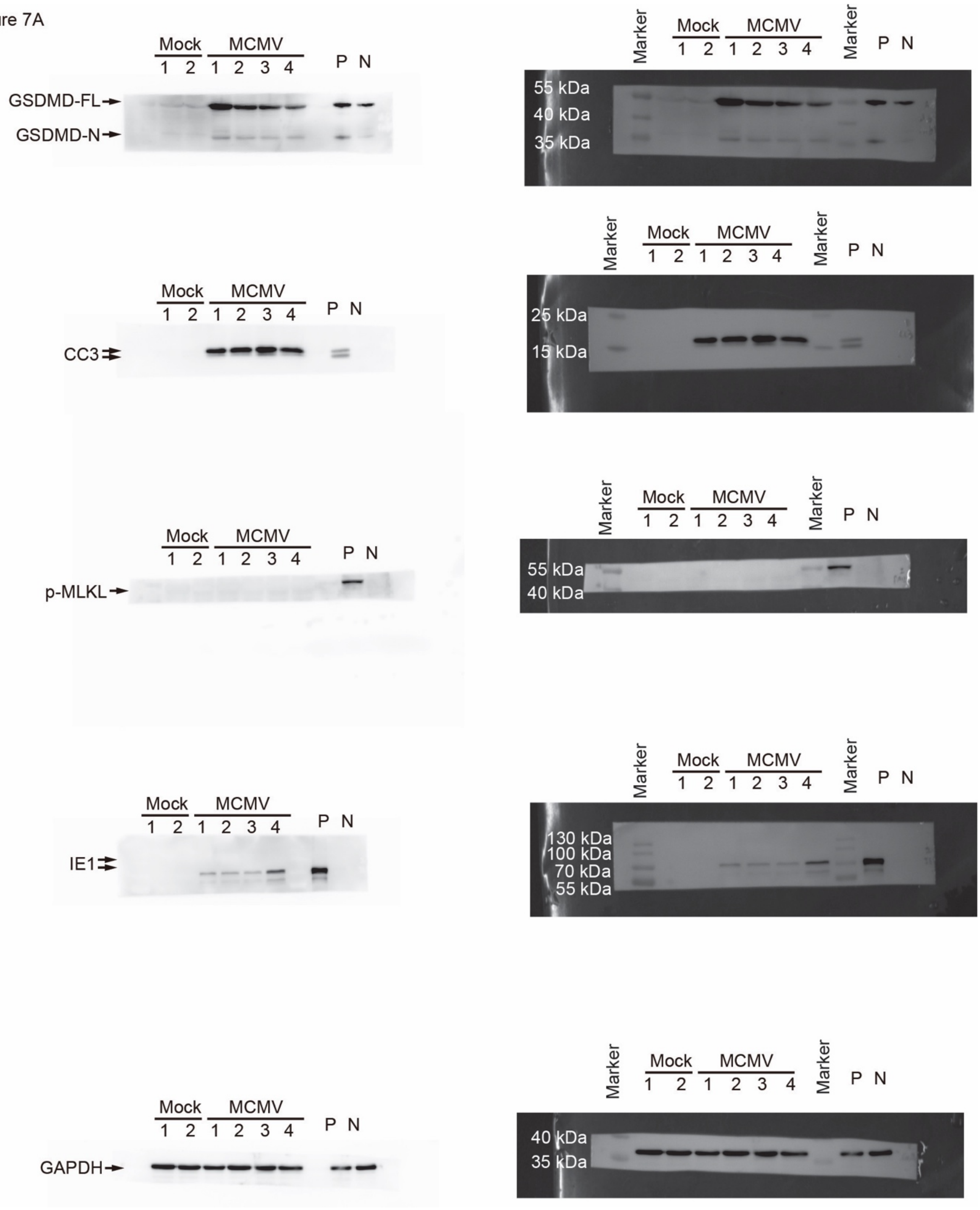

Mock
MCMV
1 2 1 2 3 4
P N
GSDMD-FL
GSDMD-N
Marker
55 kDa
40 kDa
35 kDa
CC3
25 kDa
15 kDa
p-MLKL
55 kDa
40 kDa
IE1
130 kDa
100 kDa
70 kDa
55 kDa
GAPDH
40 kDa
35 kDa

Figure 7A
P14

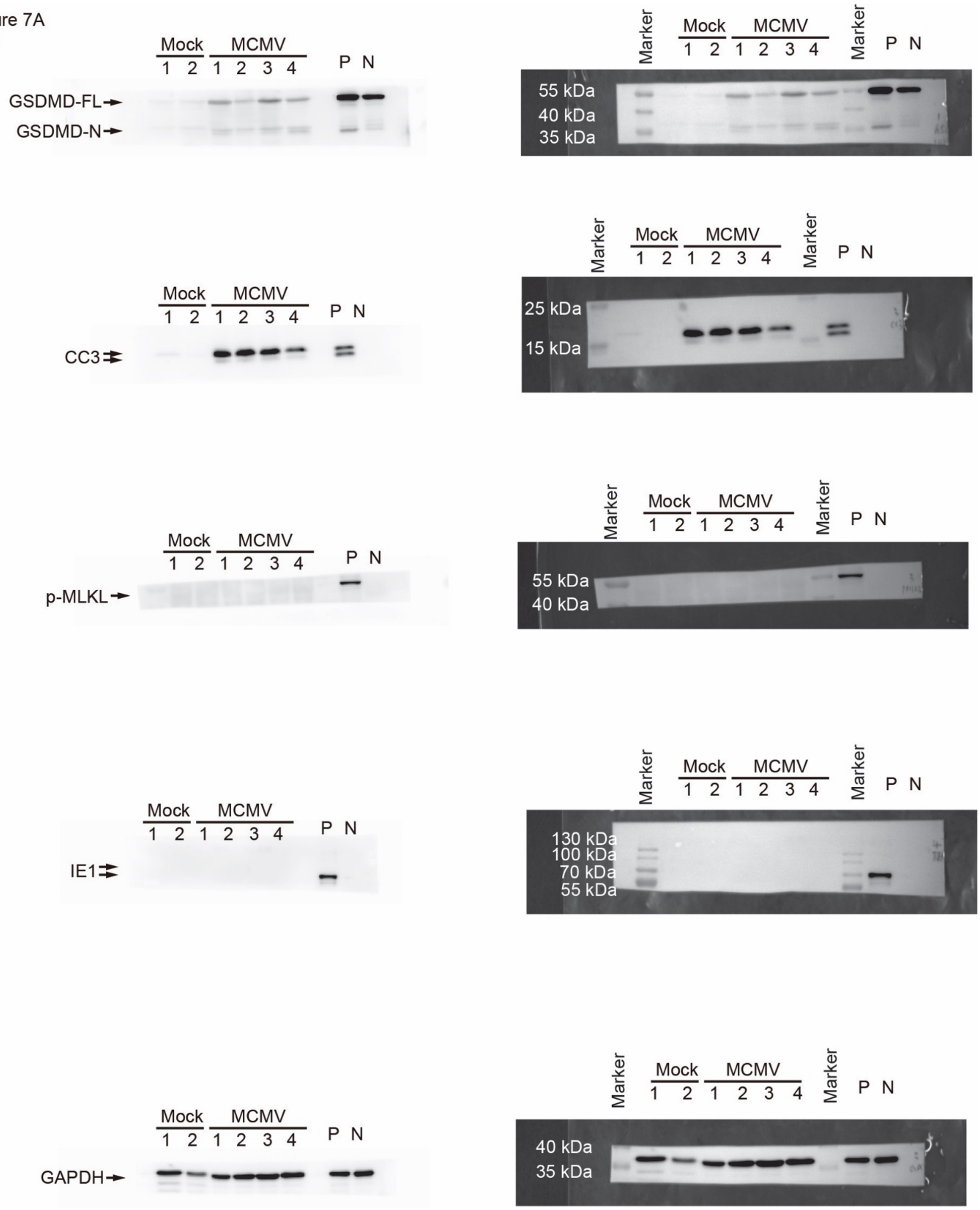

Figure 7A
P21

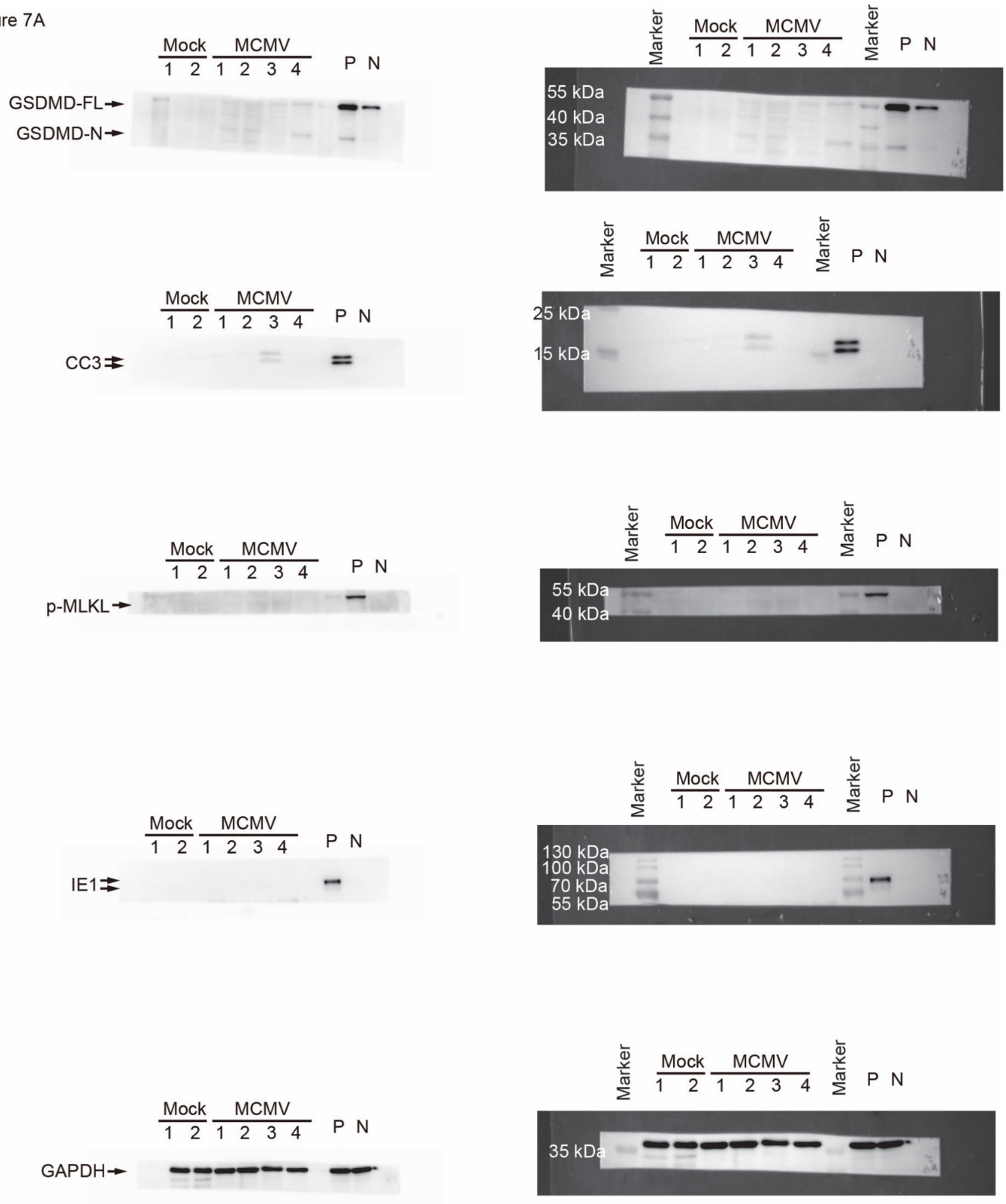

Figure 7A
P28

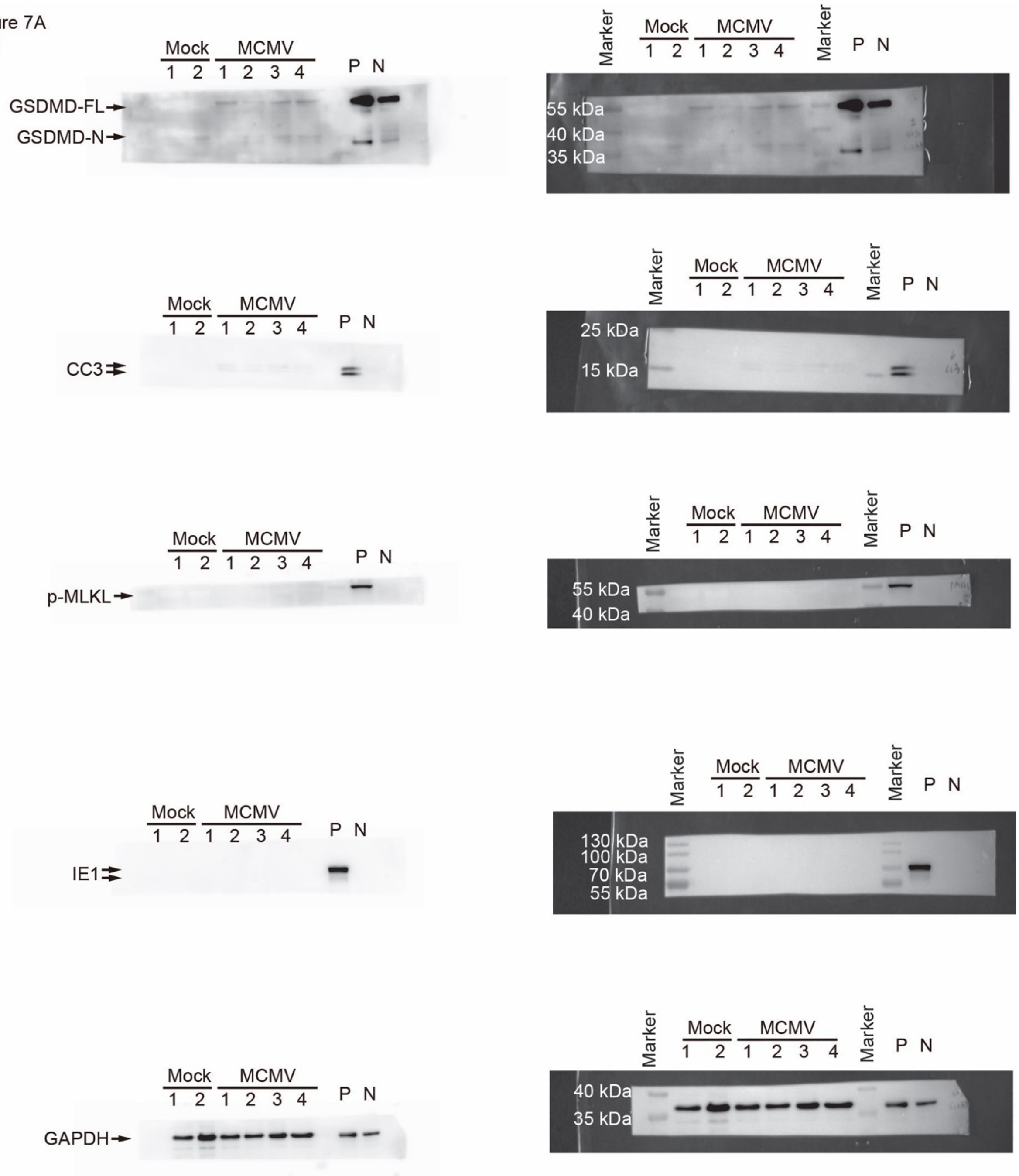

Figure 9E
P7

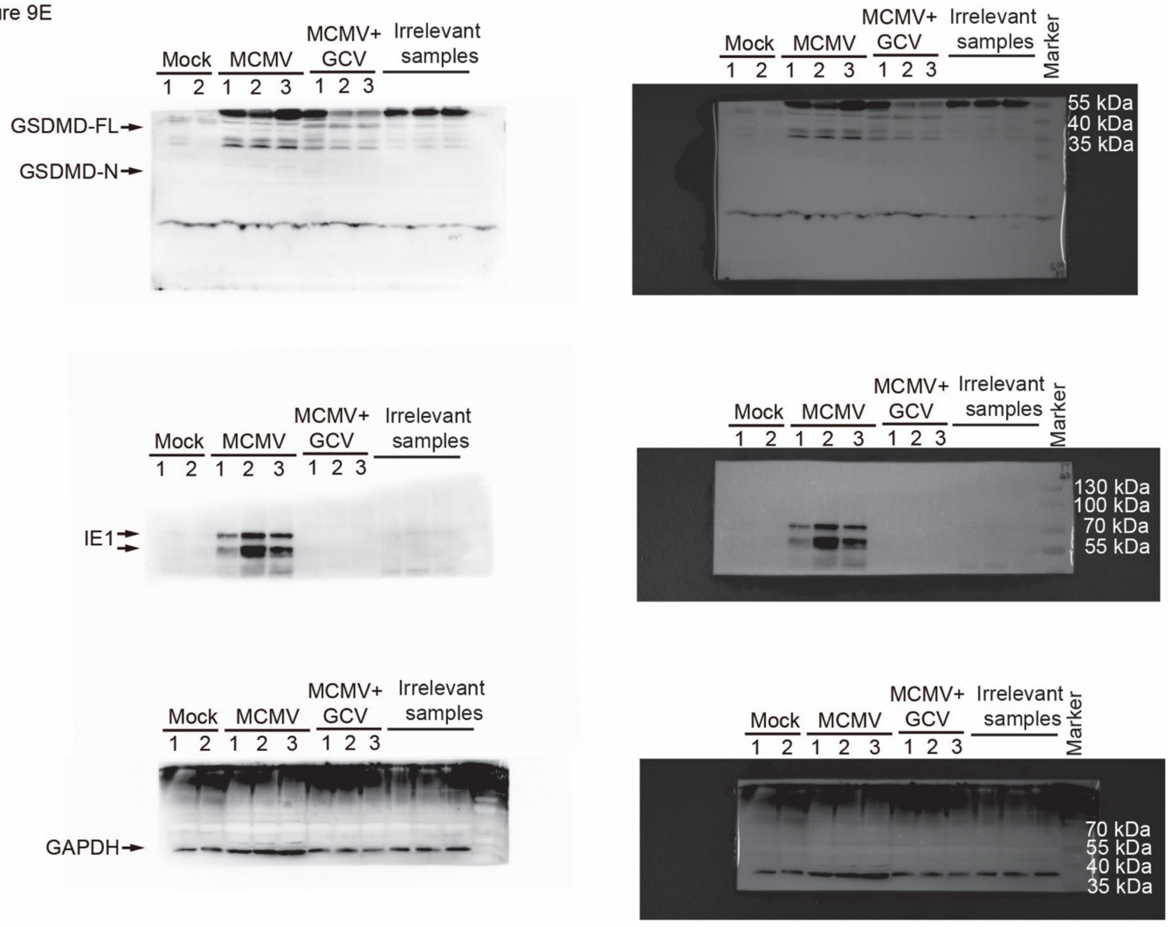
Mock
MCMV
MCMV+ GCV
Irrelevant samples
1 2 1 2 3 1 2 3
GSDMD-FL
GSDMD-N
Marker
55 kDa
40 kDa
35 kDa
IE1
130 kDa
100 kDa
70 kDa
55 kDa
GAPDH
70 kDa
55 kDa
40 kDa
35 kDa

Figure 9E
P14

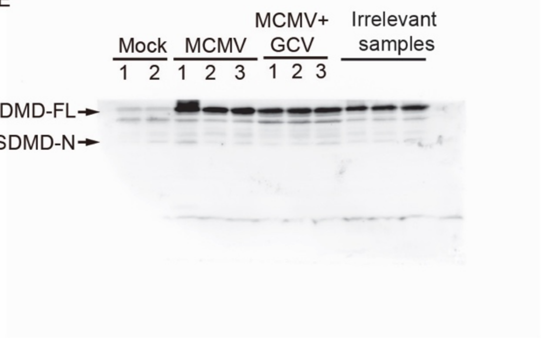

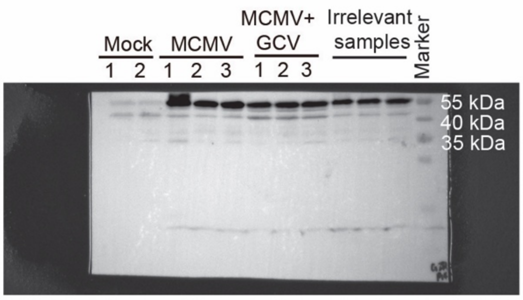

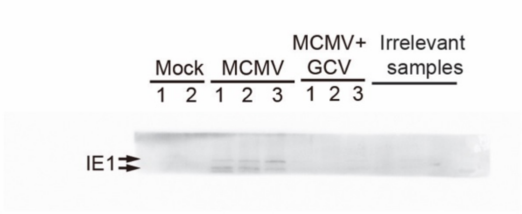

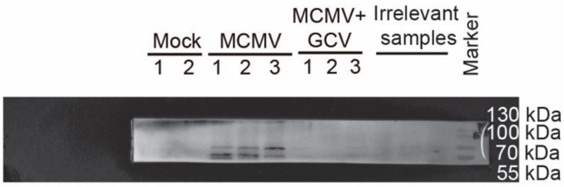

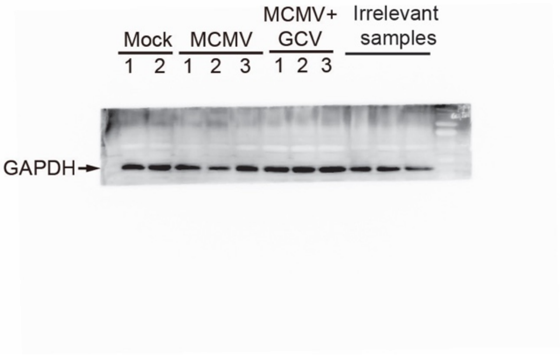

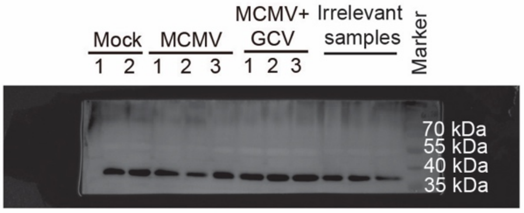

Figure 9E
P21

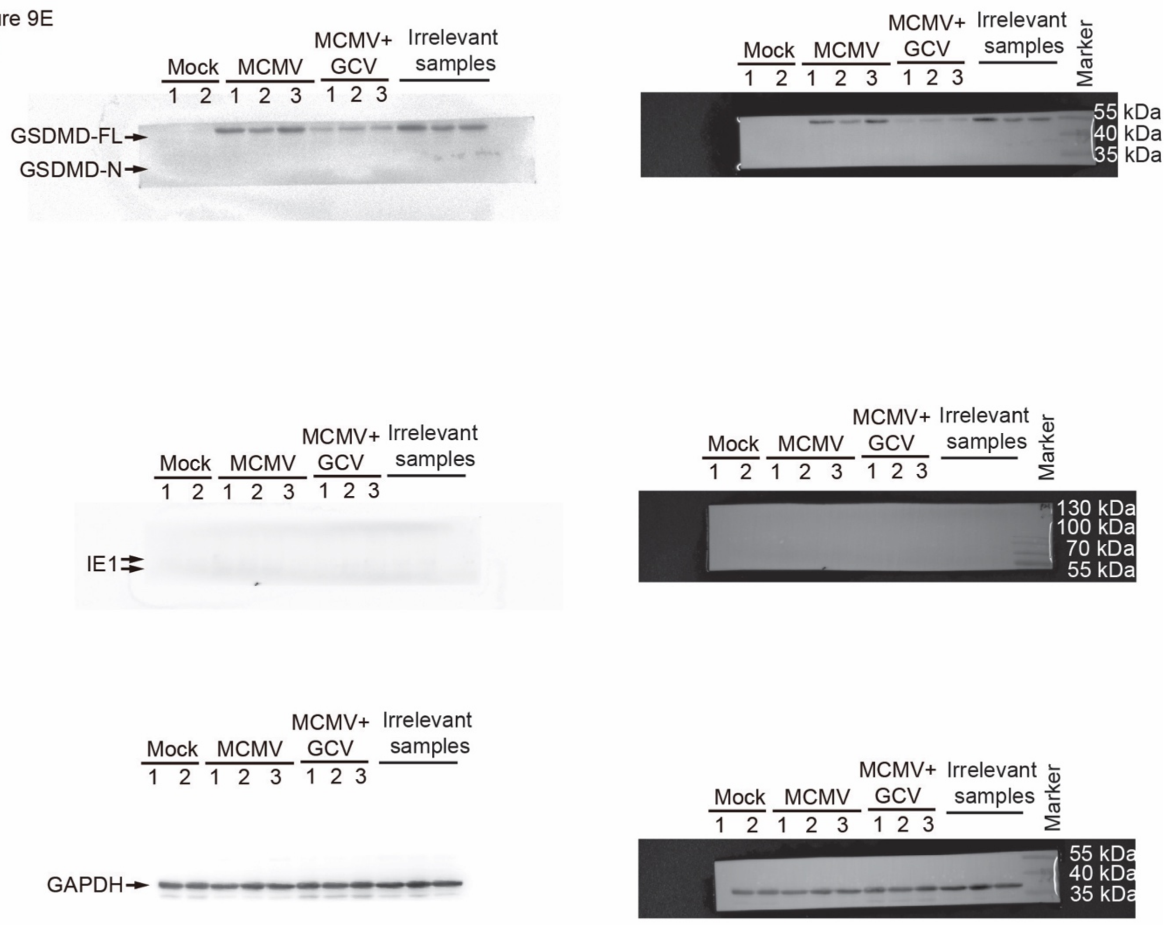

Figure 9E
P28

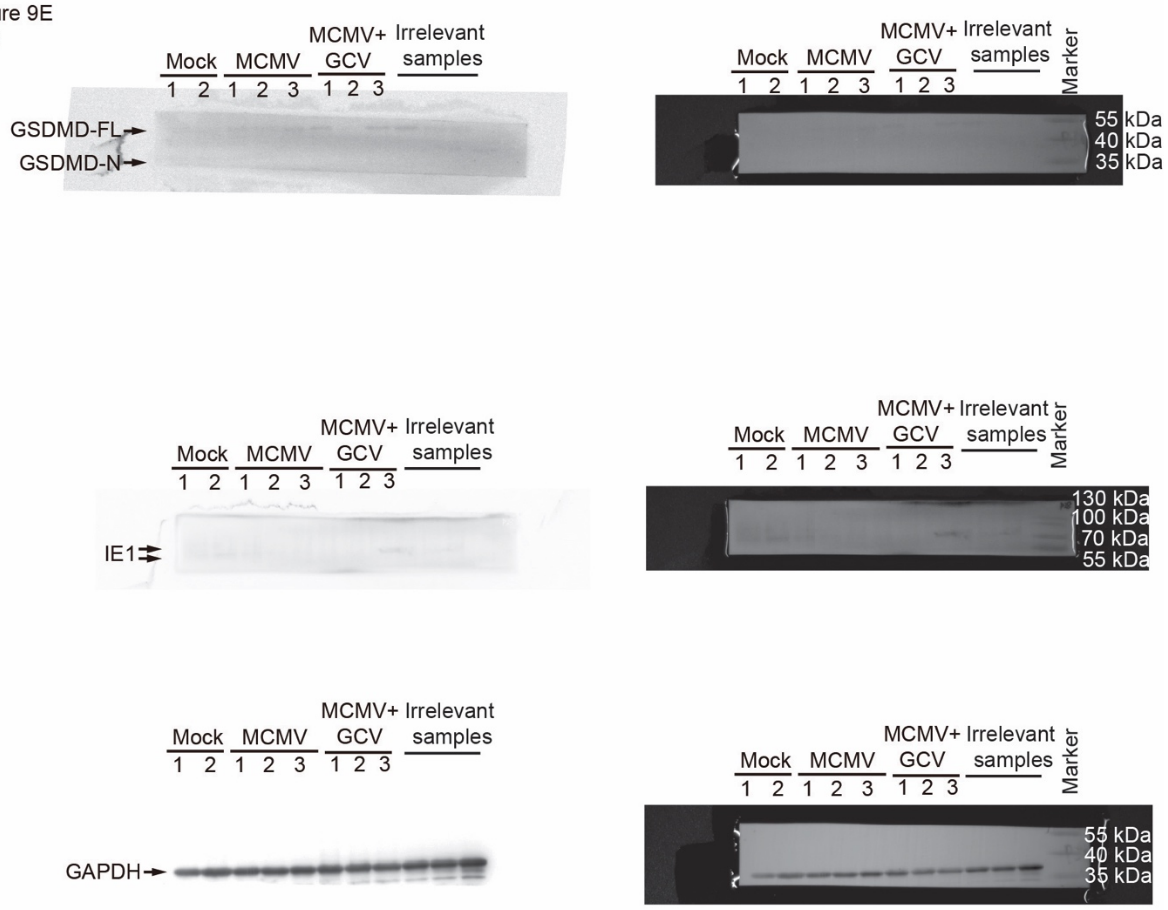

Supplement: Supplemental data [file jciinsight-7-152551-s013.pdf]
